# Comparative predictive modeling of pediatric spirometry reference equations in Jordanian children: Complex versus simple models

**DOI:** 10.1371/journal.pdig.0001746

**Published:** 2026-09-25

**Authors:** Walid Al-Qerem, Anan Jarab, Judith Eberhardt, Khalda Smairan, Yousef Mimi, Maher Khdour

**Affiliations:** 1 Department of Pharmacy, Faculty of Pharmacy, Al-Zaytoonah University of Jordan, Amman, Jordan; 2 Department of Clinical Pharmacy, Faculty of Pharmacy, Jordan University of Science and Technology, Irbid, Jordan; 3 Department of Psychology, School of Social Sciences, Humanities and Law, Teesside University, Middlesbrough, United Kingdom; 4 Department of Health Sciences, Faculty of Graduated Studies, Arab American University, Jenin, Palestine; 5 Department of Pharmacy, Faculty of Pharmacy, Al-Quds University, Jerusalem, Palestine; National Taichung University of Science and Technology, TAIWAN

## Abstract

Spirometric interpretation relies on reference equations, yet equations developed in one population or age range may not transport. Reference equations use distributional models to account for nonlinear growth, their calibration may differ across populations, it remains uncertain whether machine-learning algorithms improve prediction beyond simpler transformed regression models. This two-phase cross-sectional study compared sex-specific predictive models. Phase 1 used the same 1,576-child derivation dataset used to develop the original Jordanian GAMLSS equation (Al-Qerem equation), allowing comparison with predictive modeling strategies. Phase 2 evaluated equations in a validation sample of 1,007 healthy children aged 6–18 years. Candidate models were evaluated on the scale after back-transformation of log-outcome predictions. Models included GBM for FEV1 in both sexes, GLM for FVC in both sexes, GLM for FEV1/FVC in girls, and GBM for FEV1/FVC in boys.FEV1 and FVC were predicted more accurately than FEV1/FVC, whose explained variance from age and height remained low across model classes and established equations. In external validation, the study model had the lowest mean squared error for female FEV1 and FVC, but did not consistently outperform GLI 2012, GLI 2022, or Al-Qerem equations in boys or for FEV1/FVC. Age-stratified analyses showed elevated FEV1 and FVC below-LLN rates in boys younger than 10 years across the study, Al-Qerem, GLI 2012, and GLI 2022 equations, whereas FEV1/FVC below-LLN proportions were generally closer to nominal values. These findings support comparative predictive modeling as a useful development framework, but do not show superiority of complex machine-learning methods or readiness for clinical deployment without further calibration and external validation. Carefully chosen transformations and calibration were more important than model complexity, and the best models differed by outcome and sex.

## Author summary

Breathing tests are interpreted by comparing a child’s measured lung function with expected values for children of similar age, sex, and height. Many clinics use international equations, but these equations may not fit every population equally well. We compared simple statistical prediction models and more flexible machine-learning algorithms for Jordanian children, using the same dataset that produced a previous Jordanian reference equation and then testing the equations in a separate group of children. The main finding was practical: more complex algorithms were not consistently better. Carefully chosen transformations and calibration were more important than model complexity, and the best models differed by outcome and sex. Lung volumes were predicted more reliably than the FEV1/FVC ratio, the relationship between the air blown out in the first second and the total forced breath. These results support local validation before adopting reference equations and show that future spirometry software should combine prediction with calibration checks rather than relying only on algorithmic complexity.

## Introduction

Spirometry is the gold standard test for evaluating lung function and for identifying and monitoring respiratory conditions in children aged 6 years and older, such as asthma, which represents a substantial global health burden and is among the most common chronic diseases in childhood [1,2]. Spirometry provides objective measures of lung function, including forced expiratory volume in one second (FEV_1_), forced vital capacity (FVC), and the FEV_1_/FVC ratio [3]. To interpret these measures accurately, results are compared with predicted values derived from reference equations based on data that reflect the population’s demographic characteristics and anthropometric profile [4,5].

Conventional reference equations, including the multi-ethnic and race-neutral Global Lung Initiative (GLI) equations, are typically developed using generalized additive models for location, scale, and shape (GAMLSS) [6,7]. Although GAMLSS offers advantages, such as accounting for age-related variability and accommodating skewed spirometry distributions [8], it also has important limitations. These models are complex to implement, require substantial statistical expertise, rely heavily on spline-based transformations, and make distributional assumptions that may not hold in real-world samples. They may also require advanced statistical software, which can be difficult to access in under-resourced settings [9,10].

Global reference-equation research has therefore evolved through both multinational equations [6,7] and population-specific validation or derivation studies [11–14]. Large cross-sectional resources and recent pediatric studies from different regions [15–17] illustrate that spirometry reference modeling is best understood as a calibration and transportability problem, in which global standards, local growth patterns, and implementable analytic frameworks must be evaluated together. In digital-health settings, this algorithmic layer also requires reproducibility and software readiness, because predicted values and LLN thresholds become useful only when they can be embedded within decision-support workflows, data standards, and governance structures [18,19].

Because age, sex, height, and ethnicity strongly influence lung function, prediction equations for children and adolescents should account for these factors. Lung volumes also change nonlinearly during childhood and adolescence. For example, the FEV_1_/FVC ratio typically declines in early childhood and then increases during adolescence. As a result, these age-related trends should be considered when interpreting pulmonary function tests [20].

Predictive modeling in medicine includes a continuum from conventional regression models to flexible machine-learning algorithms. For pediatric spirometry reference equations, the central question is not whether algorithmic complexity is inherently superior, but whether a model specification accurately captures growth-related central tendency and dispersion in an external population. Because FEV1, FVC, and FEV1/FVC follow nonlinear, size-related trajectories during childhood, transformed regression models and flexible supervised learners may both be useful candidates for comparison. Therefore, the primary objective of the present study was to compare sex-specific predictive models that use age and height to estimate FEV1, FVC, and FEV1/FVC in Jordanian children and to evaluate these models against traditional GAMLSS-based reference-equation approaches. Because phase 1 of this study used the same derivation dataset as the regional Jordanian Al-Qerem GAMLSS equation [11], this design permitted a direct comparison between predictive modeling strategies and the published GAMLSS-based equation under identical derivation data. The study examined whether simple transformed generalized linear model specifications performed differently from more flexible machine-learning algorithms, and evaluated the resulting study equations against GAMLSS-based reference equations, including GLI 2012, GLI 2022 [6,7], and the regional Jordanian equation [11]. Comparisons included model fit, agreement in predicted values and lower limits of normal (LLN), z-score calibration, and differences in diagnostic classification across equations.

## Materials & methods

### Ethics statement

The phase 1 derivation data were obtained from the previously published Jordanian study, in which ethics approval and parental consent were reported [15]. Ethics approval for the phase 2 validation dataset was obtained from the Ministry of Health (Reference No.: MOH\REC\2025\267) and the Al-Zaytoonah University of Jordan Ethics Committee (Ref. No. 15/12/2024-2025). The study was conducted in accordance with the principles of the Declaration of Helsinki.

For the phase 2 validation dataset, written informed consent was obtained prior to participation. Parents completed two forms: a consent form describing the spirometry procedure and a questionnaire capturing the child’s health status and current medication use.

### Study design and participants

This cross-sectional comparative modeling study was conducted in two phases. Phase 1 used the same 1,576-child Jordanian derivation dataset that was used to develop the previously published GAMLSS-based Al-Qerem reference equations [11]. That dataset included healthy Jordanian children aged 6–17 years and was collected from schools, summer camps, and scout societies in multiple Jordanian cities, as previously described [11]. Using the same derivation data was intentional: it allowed a direct head-to-head comparison between modeling approaches without differences in sample composition being mistaken for differences in model performance. Phase 2 used an independent external-validation dataset collected between June and November 2025 from schools, public parks, summer camps in sports centers, and Quran memorization centers in multiple cities across Jordan. Children in the validation phase were eligible if they were aged 6–18 years, had no respiratory symptoms in the 2 weeks before testing, and reported no asthma-related symptoms in the past 12 months, including wheezing, nocturnal dry cough not associated with a cold or chest infection, whistling in the chest, and sneezing or a runny or blocked nose not associated with a cold or influenza.

### Measurement of anthropometric parameters

Children’s height (cm) was measured using a calibrated stadiometer, and weight (kg) was measured using a pre-calibrated digital scale, following standardized procedures to ensure measurement accuracy.

### Measurement of pulmonary function

Spirometry was performed in accordance with American Thoracic Society guidelines [16]. In phase 2, tests were conducted using a computer-based spirometer (Minispir) linked to WinSPIRO PRO software to ensure consistent measurement. Children were tested in the seated position, with a nose clip to prevent air leakage. A new disposable turbine was used for each child to reduce the risk of infection transmission.

Children were instructed to:

1. Take a deep breath.
2. Exhale as hard and as fast as possible for at least 6 seconds (at least 3 seconds for children younger than 10 years).
3. Avoid coughing or hesitating during exhalation.

Each child repeated the maneuver until three acceptable maneuvers were obtained. Between-maneuver reproducibility was assessed by comparing the two largest values for FEV_1_ and FVC, which were required to differ by no more than 0.15 L. If reproducibility criteria were not met, up to eight attempts were performed. Only acceptable maneuvers meeting reproducibility criteria were included in the final analysis.

### Statistical analysis

All statistical analyses were performed using R (R Foundation for Statistical Computing, Vienna, Austria). Continuous variables were summarized as mean (SD), and categorical variables were presented as frequency (%).

All analyses were stratified by sex. Prediction model development followed a structured comparative procedure to identify appropriate functional forms and algorithms for paediatric spirometry reference equations. Age and height were used as the primary predictors and were evaluated under alternative transformations to determine the scales that best linearized relationships with FEV1, FVC, and FEV1/FVC and stabilized residual spread.

FEV1/FVC was standardized and reported on the 0–1 ratio scale throughout the manuscript. Candidate-model performance was evaluated on the original clinical measurement scale. For log-outcome models, individual predictions were first back-transformed to the original clinical scale, and error metrics were then calculated on that scale. Candidate-model R^2^ was calculated for FEV1, FVC, and FEV1/FVC as the squared Pearson correlation between observed and out-of-fold predicted values on the original clinical measurement scale.

Three transformation frameworks were evaluated: (i) raw predictors with raw outcomes, (ii) log-transformed predictors with raw outcomes, and (iii) log-transformed predictors with log-transformed outcomes. Within each framework, five modeling approaches were applied: generalized linear models (GLM), gradient boosting machines (GBM), random forests (RF), k-nearest neighbors (KNN), and radial-kernel support vector machines (SVM). Generalized linear models occupy a dual position in this analysis. They are conventional regression models in biostatistics, but they are also widely used as interpretable supervised learning models when the objective is prediction and when model performance is evaluated through resampling, out-of-fold prediction, and external validation [21–24]. Accordingly, GLM was included in the same supervised predictive-modeling framework.

Candidate-model hyperparameters were selected within one five-fold cross-validation run without repeated resampling. The RF, SVM radial, KNN, and GBM candidates used caret default tuning grids, GLM had no tuning parameter, and the selected specification was the candidate with the lowest original-scale cross-validated RMSE after any required back-transformation.

For reproducibility, the supplementary files include reproducible R implementation code for predictive modeling, model-specification, selected hyperparameters, and scripts needed to reproduce model selection, final prediction models, z-score calculation, LLN estimation, external validation, age-stratified summaries, and ventilatory-pattern classification.

To derive clinically interpretable z-scores and lower limits of normal (LLN) for the study equations, each selected mean model was paired with a development-data sigma model for the corresponding sex-outcome combination. Individualized residual standard deviations were estimated from derivation-data out-of-fold residuals only, using the same model class and predictor scale as the mean model. The LLN was then defined using a personalized residual-SD approach based on the lower tail of the normal distribution. For raw-scale models, z-scores were calculated as the observed value minus the predicted mean divided by the individualized residual SD, and the LLN was calculated as the predicted mean minus 1.645 times that SD. For log-outcome models, z-scores and LLNs were computed on the log scale and then back-transformed to the clinical scale. For FEV1/FVC, predicted and LLN values were constrained to a physiologically plausible range.

For external validation, predicted values for FEV1, FVC, and FEV1/FVC were generated for each child using four sets of equations: GLI 2012 Caucasian, GLI 2022 global, the Jordanian Al-Qerem LMS-based equation, and the study predictive equations. For each equation, sex, and outcome, predicted values and z-scores were calculated. In the external dataset, model performance was summarized using mean squared error (MSE), with 95% confidence intervals (CIs) derived from the empirical variance of the squared residuals under an approximate normality assumption, and observed-predicted R2, defined for all three outcomes as the squared Pearson correlation between observed and predicted values on the original clinical measurement scale. Agreement between observed and predicted distributions was assessed by reporting the observed mean and SD and predicted mean and SD for FEV1, FVC, and FEV1/FVC. Calibration of z-score distributions was evaluated using their mean and SD; under ideal calibration in a healthy external sample, the mean would be 0 and the SD would be 1.

The regional Al-Qerem equation and the present study model shared the same 1,576-child derivation dataset in phase 1. This shared derivation dataset was used deliberately to make the comparison between the GAMLSS-based equation and the present predictive models a head-to-head method comparison under identical derivation data. The comparison should therefore not be interpreted as a comparison between two unrelated derivation cohorts. All equations were then evaluated in the separate 1,007-child external-validation dataset, which was not used to derive either the Al-Qerem equation or the present study model.

Formal tests of departure from the ideal reference distribution were performed. One-sample t-tests assessed whether mean z-scores differed from 0, and chi-square tests of the sample variance assessed whether the z-score SD differed from 1. Two-sided p-values were calculated, and statistical significance was set at p < 0.05. For descriptive purposes in the performance table, symbols were added next to the mean z-score and its SD to indicate statistically significant deviations from 0 and 1, respectively. Age-stratified external-validation analyses were conducted using clinically interpretable growth bands (<10, 10 to <14, and>=14 years). Within each sex and age band, training and validation sample sizes, MSE, MAE, mean z-score, z-score SD, and the proportion below the LLN were summarized for the study predictive model; age-stratified below-LLN proportions were also summarized graphically for the Al-Qerem, GLI 2012, GLI 2022, and study equations.

To further assess age- and height-related stability of FEV1/FVC lower-tail calibration, a supplementary quantile regression analysis was performed in the external-validation sample. For each sex and reference equation, FEV1/FVC z-scores were modeled as a function of age and height using quantile regression at the 5th percentile, corresponding to the lower tail of the z-score distribution. Age was entered in years and height was scaled per 10 cm. This analysis was used to assess whether LLN-relevant FEV1/FVC z-score behavior showed residual dependence on age or height after applying each equation.

Ventilatory patterns were classified according to ERS/ATS interpretive standards using equation-specific z-scores for FEV_1_/FVC and FVC. A mixed pattern was defined when both FEV_1_/FVC and FVC were below the LLN. Obstructive ventilatory impairment was defined as FEV_1_/FVC below the LLN with FVC at or above the LLN. Restrictive or nonspecific patterns were defined when FEV_1_/FVC was preserved (z ≥ −1.645) but FVC was below the LLN. A normal spirometric pattern was defined when both FEV_1_/FVC and FVC z-scores were at or above the LLN. For each equation and sex, frequencies and percentages of normal, obstructive, restrictive or nonspecific, and mixed patterns were calculated and presented graphically.

## Results

**Fig 1:**
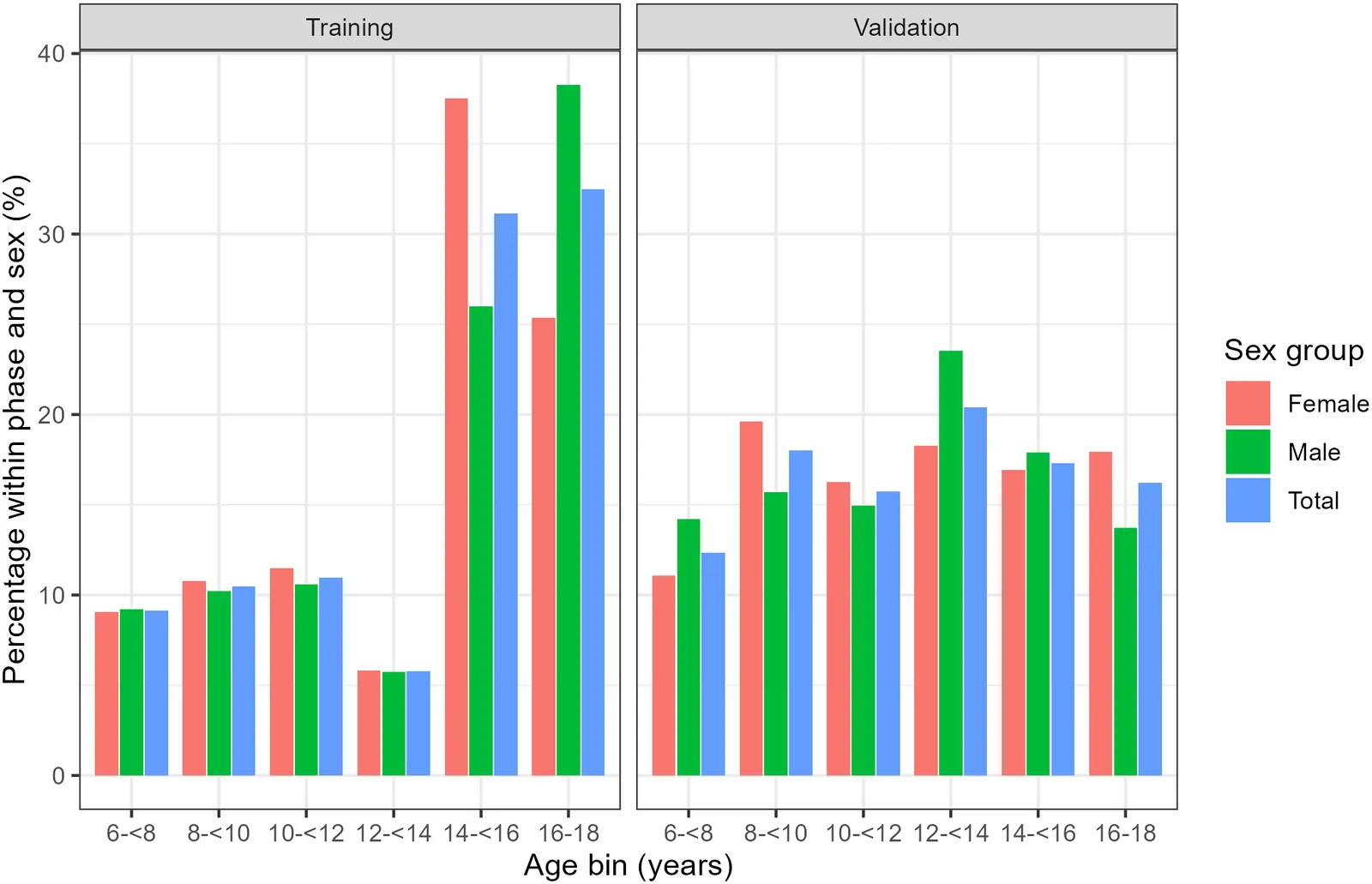
Age distribution by sex and study phase.

Sample characteristics for phases 1 and 2 are shown in Table 1 and Fig 1. In the training phase (n = 1,576), girls (n = 706) and boys (n = 870) had mean ages of 13.47 ± 3.37 and 13.76 ± 3.50 years, respectively. Boys were taller than girls on average (157.4 ± 20.3 cm vs 149.2 ± 15.4 cm) and had higher mean FEV_1_ and FVC values (boys: 3.23 ± 1.20 L and 3.68 ± 1.40 L; girls: 2.57 ± 0.75 L and 2.86 ± 0.88 L). FEV_1_/FVC ratios were high in both groups, with slightly higher values in girls than boys (0.90 ± 0.06 vs 0.88 ± 0.07). In the validation dataset (n = 1,007), mean age was 12.21 ± 3.33 years. Girls (n = 597) and boys (n = 410) had similar mean heights (147.1 ± 13.9 cm and 147.8 ± 16.5 cm), although boys again had larger lung volumes than girls for FEV_1_ (2.52 ± 0.91 L vs 2.35 ± 0.67 L) and FVC (2.90 ± 1.06 L vs 2.67 ± 0.77 L). Mean FEV_1_/FVC ratios were similar by sex in the validation sample (girls: 0.88 ± 0.06; boys: 0.87 ± 0.06).

**Table 1:** Sample characteristics of training and validation datasets.

Dataset	Sex	n	Age, years	Height, cm	FEV1, L	FVC, L	FEV1/FVC
Training	Female	706	13.47 (3.37)	149.2 (15.4)	2.57 (0.75)	2.86 (0.88)	0.90 (0.06)
Training	Male	870	13.76 (3.50)	157.4 (20.3)	3.23 (1.20)	3.68 (1.40)	0.88 (0.07)
Training	Total	1,576	13.63 (3.45)	153.7 (18.7)	2.94 (1.08)	3.31 (1.26)	0.89 (0.07)
Validation	Female	597	12.31 (3.36)	147.1 (13.9)	2.35 (0.67)	2.67 (0.77)	0.88 (0.06)
Validation	Male	410	12.06 (3.28)	147.8 (16.5)	2.52 (0.91)	2.90 (1.06)	0.87 (0.06)
Validation	Total	1,007	12.21 (3.33)	147.4 (15.0)	2.42 (0.78)	2.76 (0.90)	0.88 (0.06)

Footnote: FEV1 = forced expiratory volume in 1 second; FVC = forced vital capacity; FEV1/FVC = ratio scale (0–1).

Several alternative models were evaluated to identify the best-fitting equation for each spirometric outcome within each sex, with candidate-model metrics calculated on the original clinical scale (Tables 2 and 3). In boys, the selected model for FEV_1_ was the log-log GBM, with RMSE = 0.395 L, MAE = 0.301 L, and R^2^ = 0.892. For FVC, the selected model was the log-log GLM, with RMSE = 0.519 L, MAE = 0.393 L, and R^2^ = 0.863. In contrast, FEV_1_/FVC was weakly predicted across candidate models; the best-performing boys’ FEV_1_/FVC model was a GBM using log-transformed predictors with the raw ratio outcome, with RMSE = 0.072, MAE = 0.059, and R^2^ = 0.029. Among girls, the selected FEV_1_ model was also the log-log GBM, with RMSE = 0.314 L, MAE = 0.239 L, and R^2^ = 0.827, while the log-log GLM performed nearly identically (RMSE = 0.315 L; R^2^ = 0.826). For FVC, the selected model was a raw-scale GLM using age and height, with RMSE = 0.380 L, MAE = 0.298 L, and R^2^ = 0.811; the log-log GLM was only marginally worse (RMSE = 0.381 L; R^2^ = 0.811). For FEV_1_/FVC, the selected model was a GLM using log-transformed predictors with the raw ratio outcome, with RMSE = 0.059, MAE = 0.048, and R^2^ = 0.047.

**Table 2:** Cross-validated performance of candidate predictive models for spirometry indices in Jordanian boys in the phase 1 derivation dataset.

Algorithm	RMSE	MAE	R2	Outcome	Transformation
gbm	0.395	0.301	0.892	FEV1	Log(age,height) - > log(outcome)
glm	0.398	0.302	0.891		Log(age,height) - > log(outcome)
knn	0.408	0.315	0.885		Log(age,height) - > log(outcome)
rf	0.428	0.328	0.873		Log(age,height) - > log(outcome)
svm	0.415	0.314	0.881		Log(age,height) - > log(outcome)
gbm	0.397	0.302	0.891		Log(age,height) - > raw outcome
glm	0.439	0.346	0.866		Log(age,height) - > raw outcome
knn	0.408	0.315	0.885		Log(age,height) - > raw outcome
rf	0.430	0.328	0.872		Log(age,height) - > raw outcome
svm	0.414	0.317	0.881		Log(age,height) - > raw outcome
gbm	0.397	0.303	0.891		Raw predictors - > raw outcome
glm	0.414	0.322	0.881		Raw predictors - > raw outcome
knn	0.411	0.315	0.883		Raw predictors - > raw outcome
rf	0.430	0.328	0.873		Raw predictors - > raw outcome
svm	0.409	0.314	0.884		Raw predictors - > raw outcome
gbm	0.525	0.396	0.860	FVC	Log(age,height) - > log(outcome)
glm	0.519	0.393	0.863		Log(age,height) - > log(outcome)
knn	0.538	0.412	0.852		Log(age,height) - > log(outcome)
rf	0.574	0.437	0.833		Log(age,height) - > log(outcome)
svm	0.547	0.412	0.848		Log(age,height) - > log(outcome)
gbm	0.527	0.402	0.858		Log(age,height) - > raw outcome
glm	0.556	0.434	0.842		Log(age,height) - > raw outcome
knn	0.538	0.415	0.852		Log(age,height) - > raw outcome
rf	0.576	0.440	0.832		Log(age,height) - > raw outcome
svm	0.546	0.412	0.849		Log(age,height) - > raw outcome
gbm	0.527	0.401	0.858		Raw predictors - > raw outcome
glm	0.534	0.413	0.854		Raw predictors - > raw outcome
knn	0.544	0.416	0.849		Raw predictors - > raw outcome
rf	0.575	0.439	0.833		Raw predictors - > raw outcome
svm	0.539	0.407	0.853		Raw predictors - > raw outcome
gbm	0.073	0.060	0.024	FEV1/FVC	Log(age,height) - > log(outcome)
glm	0.073	0.060	0.005		Log(age,height) - > log(outcome)
knn	0.075	0.061	0.015		Log(age,height) - > log(outcome)
rf	0.079	0.065	0.007		Log(age,height) - > log(outcome)
svm	0.073	0.060	0.018		Log(age,height) - > log(outcome)
gbm	0.072	0.059	0.029		Log(age,height) - > raw outcome
glm	0.073	0.060	0.005		Log(age,height) - > raw outcome
knn	0.075	0.061	0.016		Log(age,height) - > raw outcome
rf	0.079	0.064	0.007		Log(age,height) - > raw outcome
svm	0.073	0.060	0.017		Log(age,height) - > raw outcome
gbm	0.072	0.059	0.028		Raw predictors - > raw outcome
glm	0.073	0.060	0.013		Raw predictors - > raw outcome
knn	0.076	0.062	0.009		Raw predictors - > raw outcome
rf	0.079	0.065	0.007		Raw predictors - > raw outcome
svm	0.073	0.060	0.016		Raw predictors - > raw outcome

Footnote: RMSE = root-mean-square error; MAE = mean absolute error; R2 = squared Pearson correlation between observed and out-of-fold predicted values on the original clinical measurement scale; GLM = generalized linear model; RF = random forest; SVM = support vector machine with radial kernel; KNN = k-nearest neighbors; GBM = gradient boosting machine. Candidate models were evaluated using one 5-fold cross-validation run without repeats. RF, SVM radial, KNN, and GBM used caret default tuning grids, and GLM had no tuning parameter. RMSE, MAE, and R2 values were calculated on the original clinical measurement scale for all candidate models. For models fitted to log-transformed outcomes, out-of-fold predictions were back-transformed before performance metrics were calculated. FEV1 and FVC are expressed in liters, and FEV1/FVC is expressed on the 0–1 ratio scale. Transformations refer to combinations of raw or log-transformed predictors (age, height) and raw or log-transformed outcomes.

**Table 3:** Cross-validated performance of candidate predictive models for spirometric indices in Jordanian girls in the phase 1 derivation dataset.

Algorithm	RMSE	MAE	R2	Outcome	Transformation
gbm	0.314	0.239	0.827	FEV1	Log(age,height) - > log(outcome)
glm	0.315	0.239	0.826		Log(age,height) - > log(outcome)
knn	0.330	0.252	0.809		Log(age,height) - > log(outcome)
rf	0.342	0.262	0.795		Log(age,height) - > log(outcome)
svm	0.323	0.247	0.817		Log(age,height) - > log(outcome)
gbm	0.315	0.240	0.826		Log(age,height) - > raw outcome
glm	0.325	0.253	0.814		Log(age,height) - > raw outcome
knn	0.329	0.252	0.810		Log(age,height) - > raw outcome
rf	0.342	0.262	0.795		Log(age,height) - > raw outcome
svm	0.321	0.245	0.819		Log(age,height) - > raw outcome
gbm	0.315	0.240	0.825		Raw predictors - > raw outcome
glm	0.316	0.242	0.825		Raw predictors - > raw outcome
knn	0.324	0.248	0.816		Raw predictors - > raw outcome
rf	0.342	0.261	0.795		Raw predictors - > raw outcome
svm	0.319	0.243	0.821		Raw predictors - > raw outcome
gbm	0.385	0.300	0.807	FVC	Log(age,height) - > log(outcome)
glm	0.381	0.295	0.811		Log(age,height) - > log(outcome)
knn	0.401	0.309	0.790		Log(age,height) - > log(outcome)
rf	0.423	0.324	0.768		Log(age,height) - > log(outcome)
svm	0.398	0.310	0.794		Log(age,height) - > log(outcome)
gbm	0.385	0.300	0.806		Log(age,height) - > raw outcome
glm	0.390	0.311	0.801		Log(age,height) - > raw outcome
knn	0.401	0.310	0.790		Log(age,height) - > raw outcome
rf	0.422	0.326	0.769		Log(age,height) - > raw outcome
svm	0.403	0.313	0.790		Log(age,height) - > raw outcome
gbm	0.385	0.300	0.807		Raw predictors - > raw outcome
glm	0.380	0.298	0.811		Raw predictors - > raw outcome
knn	0.391	0.304	0.801		Raw predictors - > raw outcome
rf	0.422	0.325	0.769		Raw predictors - > raw outcome
svm	0.397	0.308	0.795		Raw predictors - > raw outcome
gbm	0.059	0.049	0.046	FEV1/FVC	Log(age,height) - > log(outcome)
glm	0.059	0.048	0.048		Log(age,height) - > log(outcome)
knn	0.062	0.051	0.018		Log(age,height) - > log(outcome)
rf	0.064	0.052	0.014		Log(age,height) - > log(outcome)
svm	0.060	0.049	0.032		Log(age,height) - > log(outcome)
gbm	0.059	0.048	0.047		Log(age,height) - > raw outcome
glm	0.059	0.048	0.047		Log(age,height) - > raw outcome
knn	0.062	0.051	0.018		Log(age,height) - > raw outcome
rf	0.064	0.052	0.014		Log(age,height) - > raw outcome
svm	0.060	0.049	0.034		Log(age,height) - > raw outcome
gbm	0.059	0.049	0.051		Raw predictors - > raw outcome
glm	0.059	0.048	0.045		Raw predictors - > raw outcome
knn	0.061	0.050	0.029		Raw predictors - > raw outcome
rf	0.065	0.053	0.011		Raw predictors - > raw outcome
svm	0.060	0.049	0.038		Raw predictors - > raw outcome

Footnote: RMSE = root-mean-square error; MAE = mean absolute error; R2 = squared Pearson correlation between observed and out-of-fold predicted values on the original clinical measurement scale; GLM = generalized linear model; RF = random forest; SVM = support vector machine with radial kernel; KNN = k-nearest neighbors; GBM = gradient boosting machine. Candidate models were evaluated using one 5-fold cross-validation run without repeats. RF, SVM radial, KNN, and GBM used caret default tuning grids, and GLM had no tuning parameter. RMSE, MAE, and R2 values were calculated on the original clinical measurement scale for all candidate models. For models fitted to log-transformed outcomes, out-of-fold predictions were back-transformed before performance metrics were calculated. FEV1 and FVC are expressed in liters, and FEV1/FVC is expressed on the 0–1 ratio scale. Transformations refer to combinations of raw or log-transformed predictors (age, height) and raw or log-transformed outcomes.

**Fig 2:**
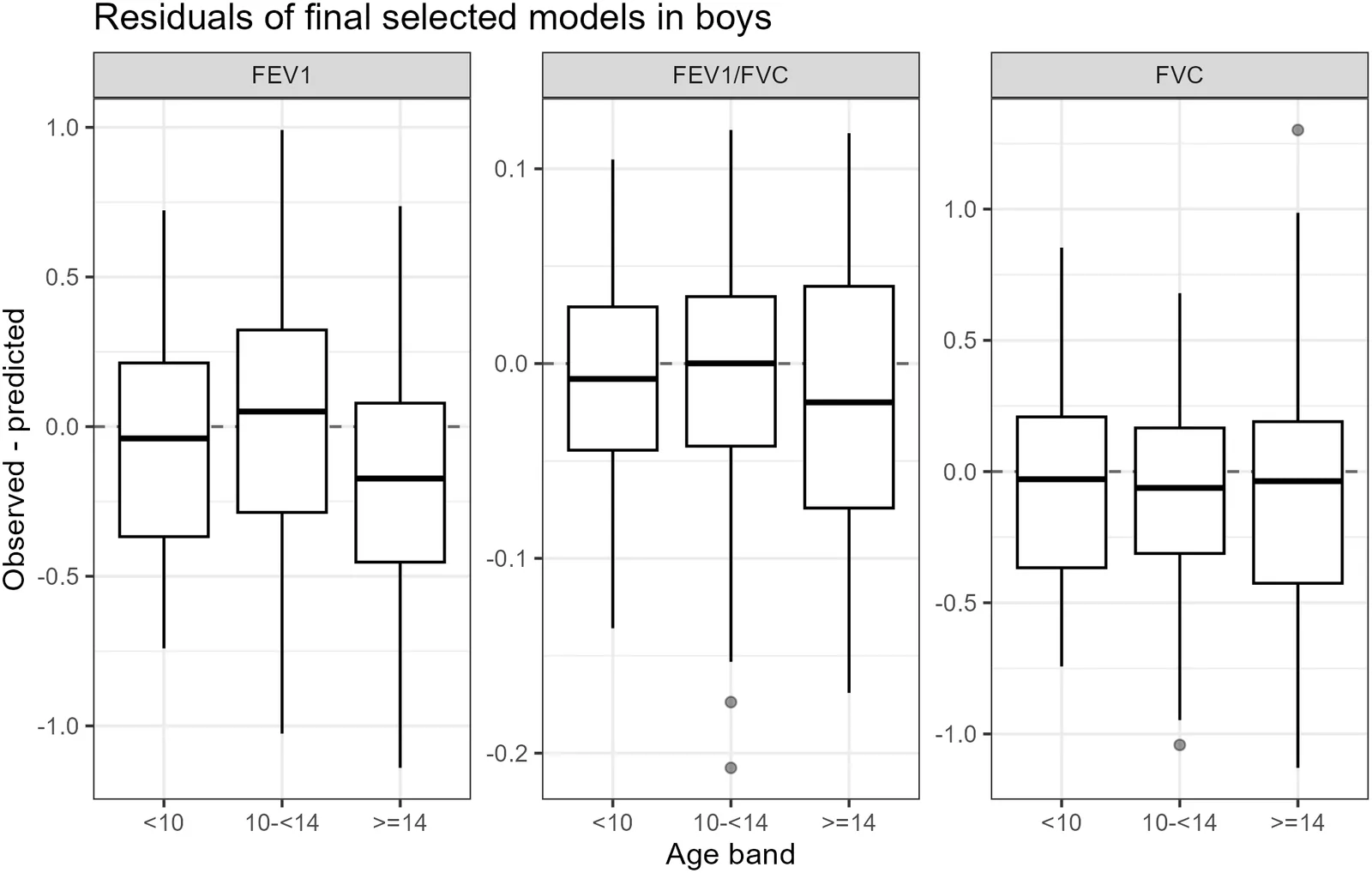
Residuals of selected models by age band in boys.

**Fig 3:**
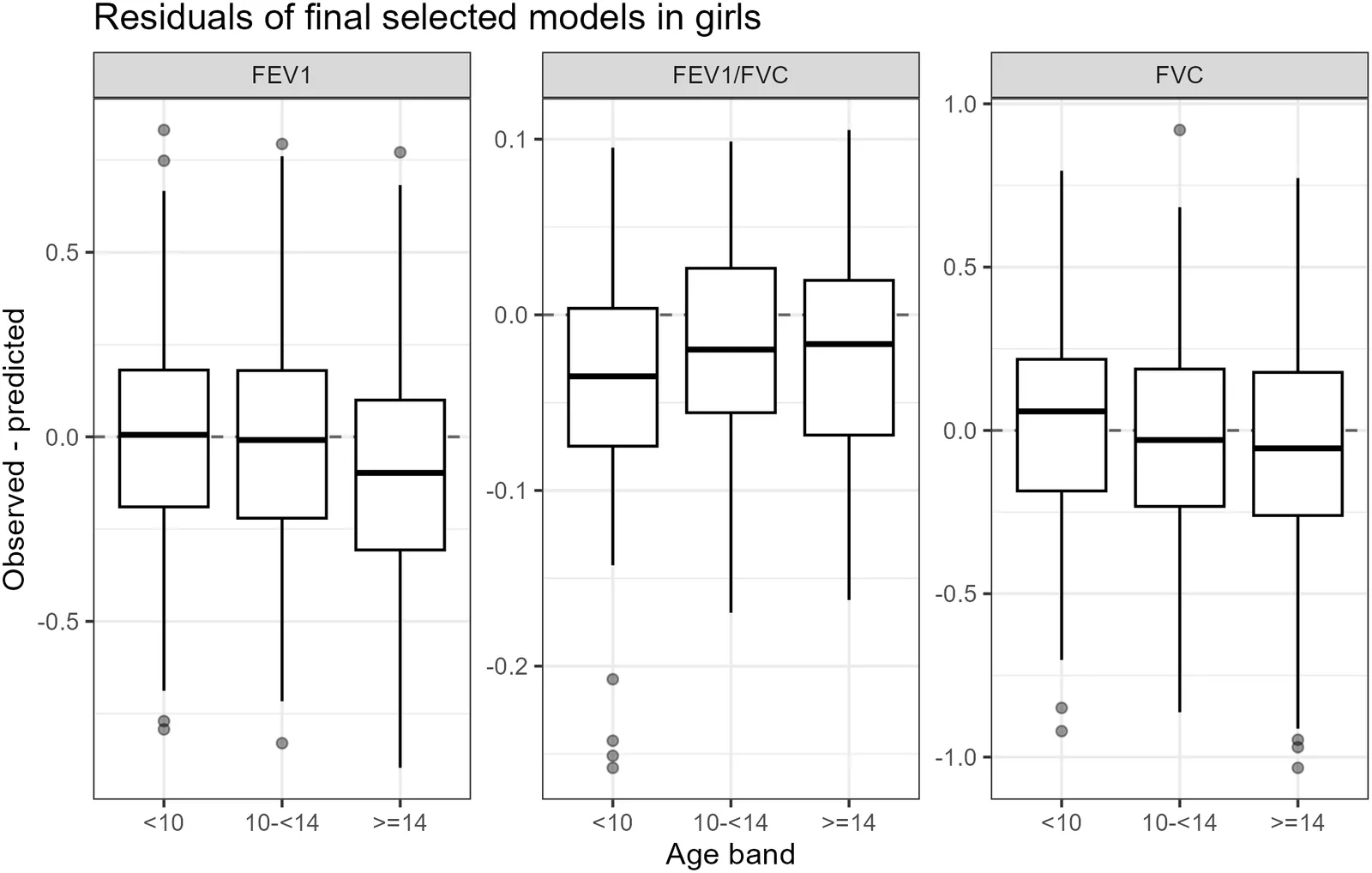
Residuals of selected models by age band in girls.

Residual plots were examined to assess model suitability Figs 2 and 3. For both sexes, residuals from the selected FEV_1_ and FVC models were generally centered around zero across age groups, although dispersion increased among older boys. Residuals for FEV_1_/FVC were wider and more heterogeneous than those for volume outcomes, particularly across age bins in boys, consistent with the low explained variance observed for the ratio outcome.

In the external validation dataset, the study predictive model did not show uniform superiority over the three established reference equations across sexes and spirometric outcomes (Table 4). Among females, the study model for FEV_1_ yielded the lowest MSE (0.095; 95% CI, 0.085 to 0.106), compared with Al-Qerem (0.114), GLI 2012 Caucasian (0.119), and GLI 2022 (0.112). Its observed-predicted R^2^ was also highest at 0.798, compared with 0.765 for Al-Qerem, 0.769 for GLI 2012, and 0.758 for GLI 2022. The observed mean FEV_1_ in females was 2.35 ± 0.67 L and was closely approximated by the study-model prediction of 2.38 ± 0.65 L. For female FVC, the study model again had the lowest MSE (0.110; 95% CI, 0.097 to 0.122), compared with Al-Qerem (0.118), GLI 2012 (0.119), and GLI 2022 (0.126), while observed-predicted R^2^ values were similar across equations (study model: 0.817; Al-Qerem: 0.811; GLI 2012: 0.813; GLI 2022: 0.800). Study-model z-score SDs were 1.30 for female FEV_1_ and 0.91 for female FVC, indicating moderate departure from the ideal reference distribution for FEV_1_ but acceptable dispersion for FVC. For female FEV_1_/FVC, the study model had an MSE of 0.004, similar to the comparator equations, but R^2^ remained very low across all equations, ranging from 0.0017 to 0.0079, with a study-model R^2^ of 0.0026.

**Table 4:** Model performance and z-score tests across reference equations and the study predictive model.

Sex	Outcome	Model	MSE, 95% CI	Observed mean + /- SD	Predicted mean + /- SD	Z-score (tests)	R2
Female	FEV1	Al-Qerem	0.114 [0.101, 0.127]	2.35 + /- 0.67	2.39 + /- 0.66	-0.19* (1.29†)	0.7647
		GLI 2012 Caucasian	0.119 [0.105, 0.134]		2.42 + /- 0.68	-0.21* (1.24†)	0.7694
		GLI 2022	0.112 [0.099, 0.125]		2.30 + /- 0.61	0.17* (1.18†)	0.7581
		Study predictive model	0.095 [0.085, 0.106]		2.38 + /- 0.65	-0.23* (1.30†)	0.7978
	FVC	Al-Qerem	0.118 [0.104, 0.131]	2.67 + /- 0.77	2.65 + /- 0.77	0.04 (1.10†)	0.8108
		GLI 2012 Caucasian	0.119 [0.105, 0.134]		2.73 + /- 0.77	-0.13* (1.11†)	0.8129
		GLI 2022	0.126 [0.112, 0.139]		2.58 + /- 0.69	0.27* (1.05)	0.8004
		Study predictive model	0.110 [0.097, 0.122]		2.70 + /- 0.74	-0.05 (0.91†)	0.8172
	FEV1/FVC	Al-Qerem	0.004 [0.004, 0.005]	0.88 + /- 0.06	0.91 + /- 0.01	-0.45* (0.91†)	0.0017
		GLI 2012 Caucasian	0.003 [0.003, 0.004]		0.90 + /- 0.01	-0.21* (0.94†)	0.0070
		GLI 2022	0.004 [0.003, 0.004]		0.90 + /- 0.01	-0.24* (0.95)	0.0079
		Study predictive model	0.004 [0.003, 0.004]		0.90 + /- 0.01	-0.39* (0.88†)	0.0026
Male	FEV1	Al-Qerem	0.147 [0.129, 0.164]	2.52 + /- 0.91	2.59 + /- 0.88	-0.32* (1.37†)	0.8312
		GLI 2012 Caucasian	0.148 [0.131, 0.166]		2.58 + /- 0.87	-0.20* (1.44†)	0.8272
		GLI 2022	0.145 [0.128, 0.162]		2.46 + /- 0.78	0.19* (1.40†)	0.8317
		Study predictive model	0.157 [0.138, 0.177]		2.58 + /- 0.88	-0.30* (1.35†)	0.8189
	FVC	Al-Qerem	0.154 [0.134, 0.174]	2.90 + /- 1.06	2.94 + /- 1.01	-0.27* (1.27†)	0.8651
		GLI 2012 Caucasian	0.163 [0.142, 0.183]		2.99 + /- 1.02	-0.27* (1.30†)	0.8628
		GLI 2022	0.163 [0.141, 0.185]		2.82 + /- 0.92	0.14* (1.25†)	0.8632
		Study predictive model	0.156 [0.136, 0.176]		2.96 + /- 1.01	-0.24* (1.11†)	0.8651
	FEV1/FVC	Al-Qerem	0.004 [0.003, 0.004]	0.87 + /- 0.06	0.89 + /- 0.01	-0.16* (0.84†)	0.0010
		GLI 2012 Caucasian	0.004 [0.003, 0.004]		0.87 + /- 0.01	0.09 (1.00)	0.0021
		GLI 2022	0.004 [0.003, 0.004]		0.87 + /- 0.01	0.04 (1.00)	0.0030
		Study predictive model	0.004 [0.003, 0.004]		0.88 + /- 0.01	-0.13* (0.84†)	0.0003

Note. R2 = squared Pearson correlation between observed and predicted values on the original clinical measurement scale. * mean z differed from 0 (p < .05); †)z-score SD differed from 1 (p < .05). FEV1 = forced expiratory volume in 1 second; FVC = forced vital capacity.

Among males, the study model did not outperform the established equations for FEV_1_ or FVC. For FEV_1_, MSE was 0.157 for the study model, compared with 0.147 for Al-Qerem, 0.148 for GLI 2012, and 0.145 for GLI 2022; observed-predicted R^2^ was 0.819 for the study model, compared with 0.831, 0.827, and 0.832 for Al-Qerem, GLI 2012, and GLI 2022, respectively. For male FVC, the study-model MSE was 0.156, slightly higher than Al-Qerem (0.154) but lower than GLI 2012 and GLI 2022, both 0.163, while R^2^ values were nearly identical across equations (study model: 0.865; Al-Qerem: 0.865; GLI 2012: 0.863; GLI 2022: 0.863). For male FEV_1_/FVC, MSE was similar across equation sets, the study-model z-score SD was 0.84, and R^2^ remained close to zero for all equations, ranging from 0.0003 to 0.0030. Overall, observed-predicted R^2^ values showed a clear separation between volume outcomes and FEV_1_/FVC: age and height explained substantial variation in FEV_1_ and FVC but little individual-level variation in the ratio, a pattern shared by the study model and established reference-equation frameworks.

**Fig 4:**
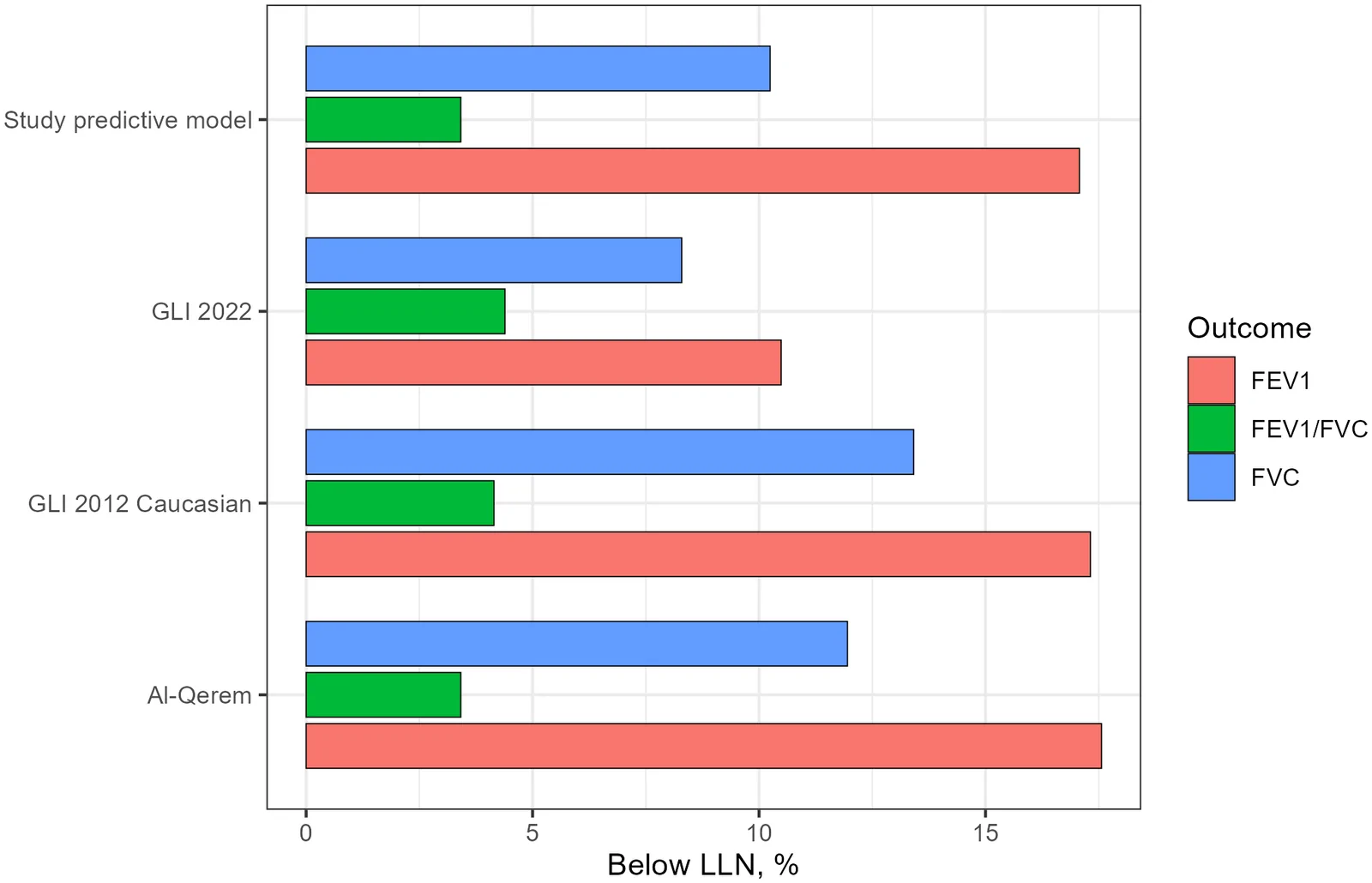
Proportion below the lower limit of normal (z < -1.645) by equation in boys.

**Fig 5:**
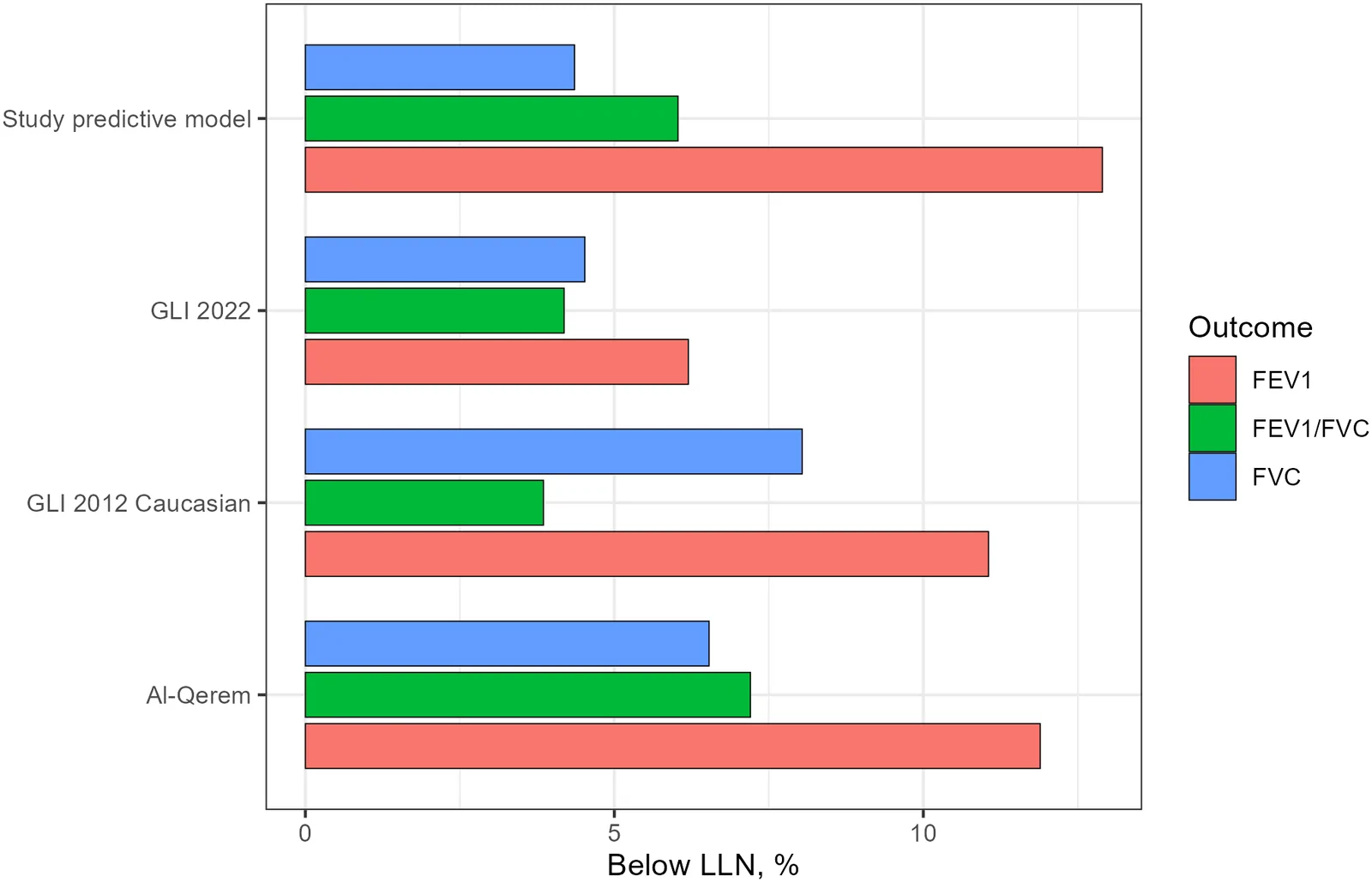
Proportion below the lower limit of normal (z < -1.645) by equation in girls.

Across equations, the proportion of children with values below the LLN (z <−1.645) varied by outcome and sex Figs 4 and 5. Using the development-derived personalized-SD LLN, the study equation classified 12.9% of females below the LLN for FEV_1_, 4.4% for FVC, and 6.0% for FEV_1_/FVC. By comparison, the GLI and Al-Qerem equations classified 6.2% to 11.9% of girls below the LLN for FEV_1_, 4.5% to 8.0% for FVC, and 3.9% to 7.2% for FEV_1_/FVC. Among males, below-LLN proportions using the study equation were 17.1% for FEV_1_, 10.2% for FVC, and 3.4% for FEV_1_/FVC. The GLI and Al-Qerem equations classified 10.5% to 17.6% of boys below the LLN for FEV_1_, 8.3% to 13.4% for FVC, and 3.4% to 4.4% for FEV_1_/FVC. Thus, the study equation produced below-LLN rates that were broadly comparable with established equations.

**Fig 6:**
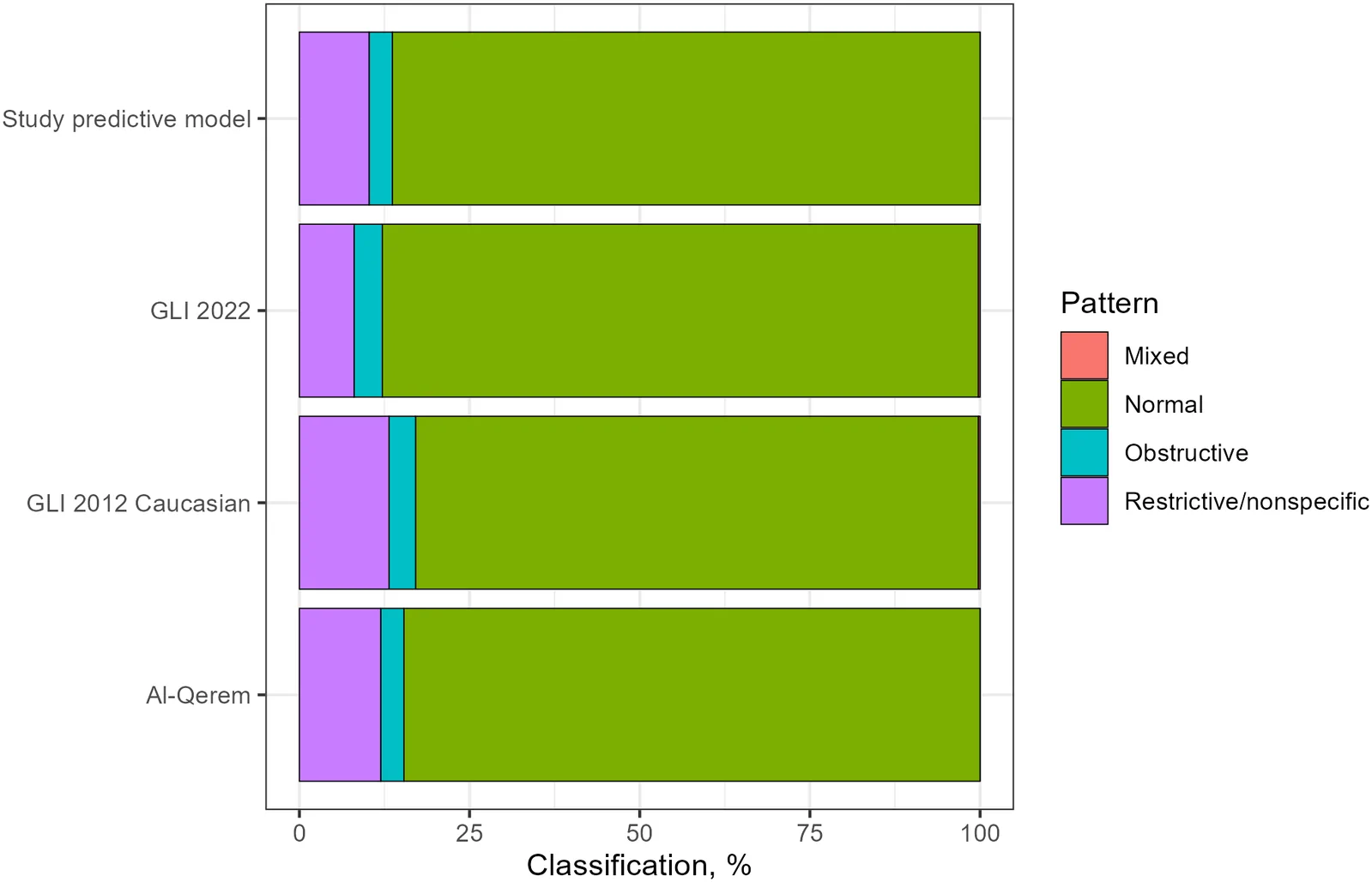
Ventilatory-pattern classification by equation in boys.

**Fig 7:**
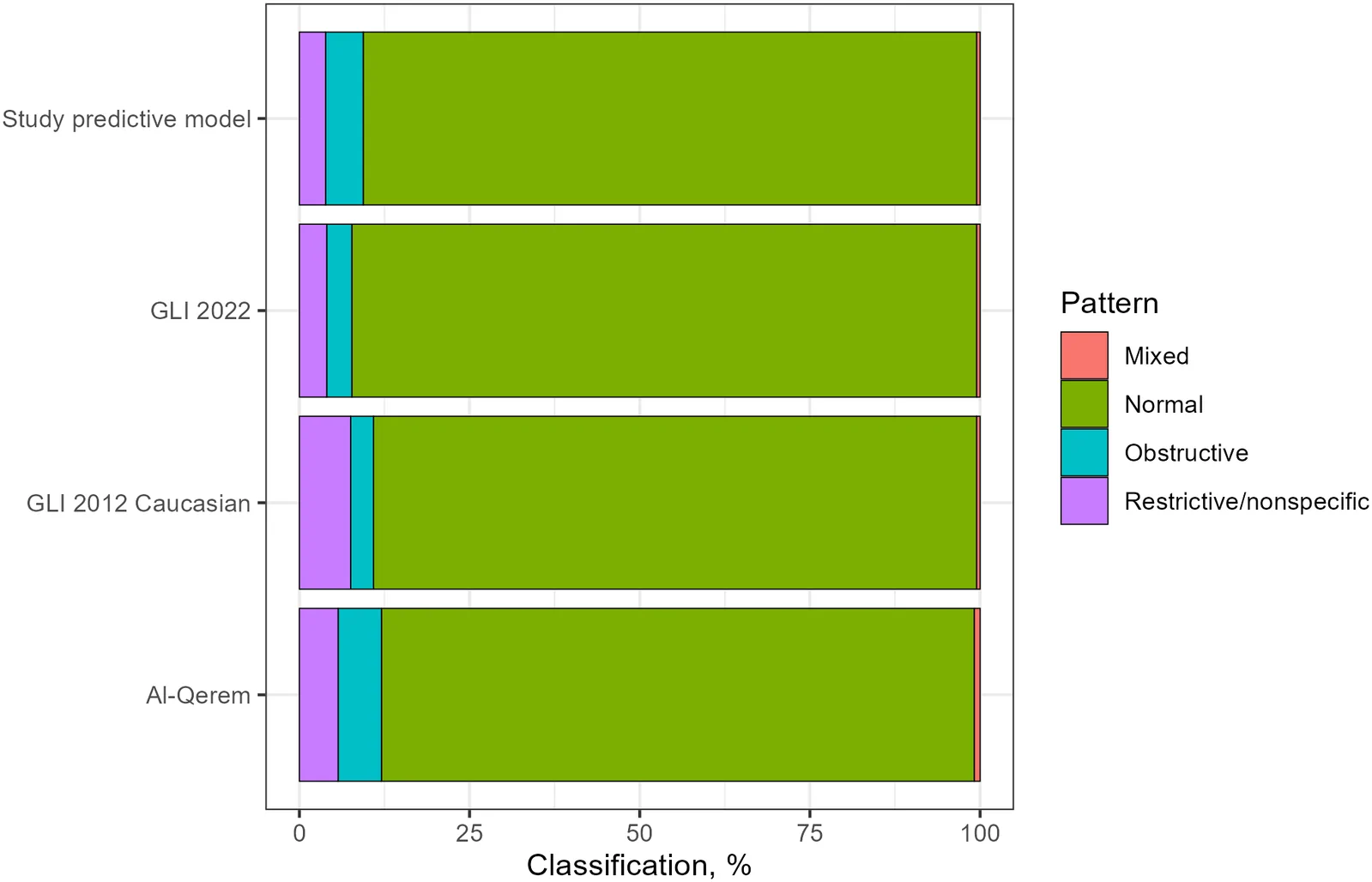
Ventilatory-pattern classification by equation in girls.

In the external validation sample, normal spirometry remained the most common ventilatory pattern across all reference equations, although the proportion classified as abnormal differed by equation set and sex Figs 6 and 7. Among females, the study equation classified 90.1% as normal, 5.5% as obstructive, 3.9% as restrictive or nonspecific, and 0.5% as mixed. By comparison, normal classification was 87.1% with Al-Qerem, 88.6% with GLI 2012 Caucasian, and 91.8% with GLI 2022. Obstructive impairment in girls was identified in 6.4% using Al-Qerem, 3.4% using GLI 2012, and 3.7% using GLI 2022, while restrictive or nonspecific patterns were 5.7%, 7.5%, and 4.0%, respectively.

Findings in males showed a similar pattern. The study model classified 86.3% of boys as normal, 3.4% as obstructive, 10.2% as restrictive or nonspecific, and 0.0% as mixed. In contrast, normal classification was 84.6% with Al-Qerem, 82.7% with GLI 2012, and 87.6% with GLI 2022. Obstructive impairment was identified in 3.4% to 4.1% using the comparator equations, restrictive or nonspecific patterns in 8.0% to 13.2%, and mixed patterns in 0.0% to 0.2%. These findings indicate that ventilatory-pattern estimates were sensitive to equation-specific mean and dispersion functions, even though normal spirometry remained the dominant classification across all frameworks.

**Fig 8:**
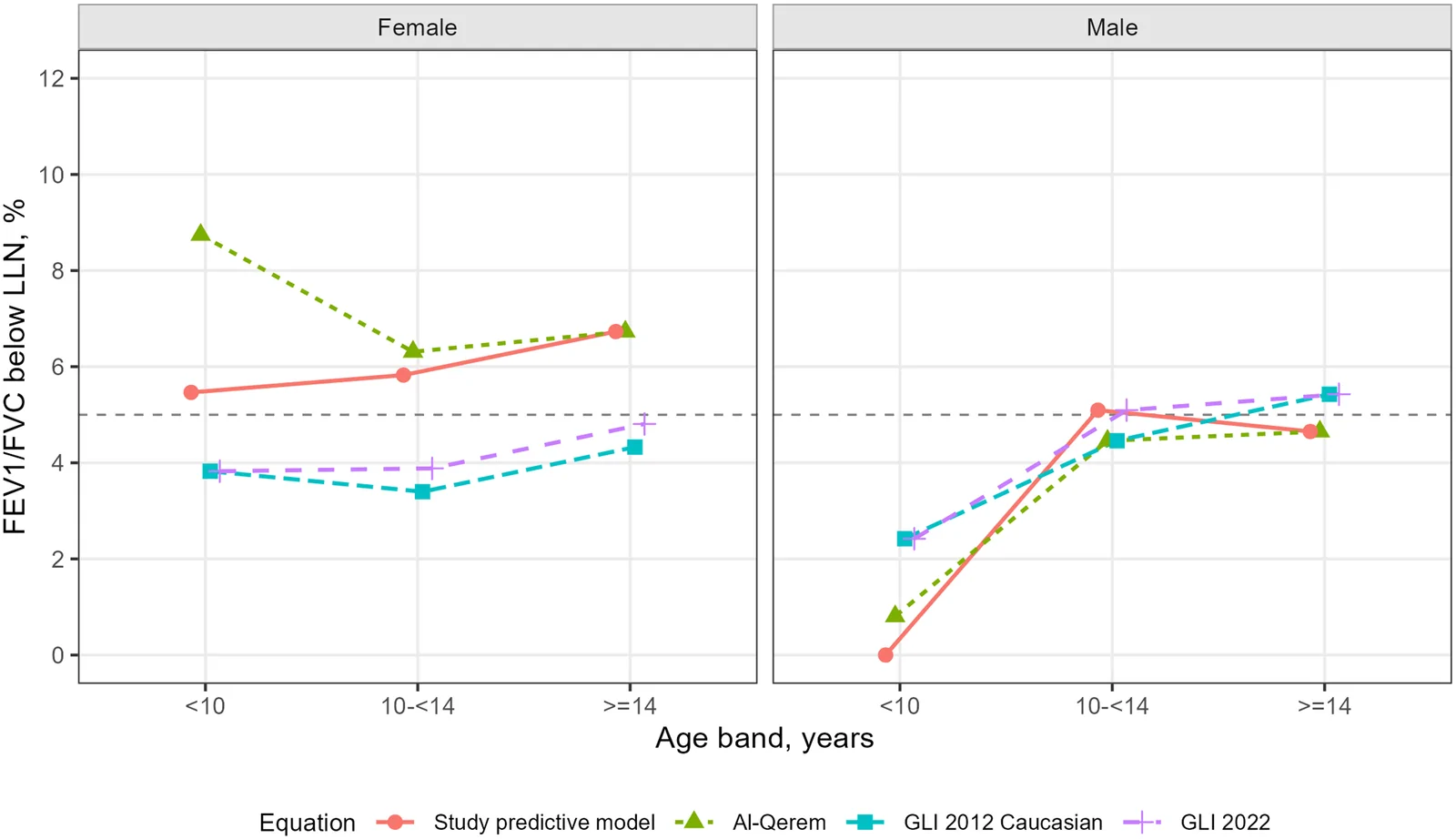
Age-stratified FEV1/FVC below-LLN proportions by equation and sex. Note. The dashed line marks the nominal 5% lower-tail expectation. Equation-specific points are horizontally offset to display closely overlapping values.

Age-stratified external validation showed variation in model performance and LLN calibration across growth bands (Table 5). With the final study models, FEV_1_ MSE among girls was 0.081, 0.096, and 0.106 in the < 10, 10 to <14, and ≥14 year bands, respectively; corresponding values among boys were 0.132, 0.149, and 0.192. For FVC, MSE values were 0.093, 0.117, and 0.117 across the three female age bands and 0.148, 0.137, and 0.186 across the corresponding male bands. In the broader equation comparison, the most pronounced age-band departure occurred for volume outcomes in boys younger than 10 years: FEV_1_ below-LLN proportions ranged from 26.6% with GLI 2022 to 32.3% with GLI 2012, and FVC below-LLN proportions ranged from 21.0% with the study and GLI 2022 equations to 29.0% with GLI 2012. In boys aged 10 to <14 years, FEV_1_ and FVC below-LLN proportions were lower across equations, ranging from 5.1% to 10.8% and 4.5% to 8.9%, respectively. In boys aged ≥14 years, the corresponding ranges were 1.6% to 16.3% for FEV_1_ and 0.8% to 3.9% for FVC. Among girls, age-stratified volume below-LLN proportions were less extreme, although FEV_1_ remained above the nominal 5% level for most equations across age bands. Comparative FEV_1_/FVC below-LLN proportions across equations are shown in Fig 8 and were generally closer to the nominal lower-tail expectation across age bands.

**Table 5:** Age-stratified external-validation performance of the study predictive model.

Sex	Age band	Training n (%)	Validation n (%)	Outcome	MSE	MAE	Mean z (SD)	Below LLN, %
Female	<10	140 (19.8)	183 (30.7)	FEV1	0.081	0.220	-0.11 (1.55)	13.1
				FVC	0.093	0.241	0.13 (1.00)	2.7
				FEV1/FVC	0.005	0.053	-0.53 (0.82)	5.5
	10- < 14	122 (17.3)	206 (34.5)	FEV1	0.096	0.250	-0.15 (1.19)	11.7
				FVC	0.117	0.271	-0.14 (0.93)	6.8
				FEV1/FVC	0.003	0.048	-0.31 (0.84)	5.8
	>=14	444 (62.9)	208 (34.8)	FEV1	0.106	0.258	-0.41 (1.14)	13.9
				FVC	0.117	0.274	-0.11 (0.77)	3.4
				FEV1/FVC	0.004	0.050	-0.34 (0.95)	6.7
Male	<10	169 (19.4)	124 (30.2)	FEV1	0.132	0.312	-0.47 (1.78)	29.0
				FVC	0.148	0.324	-0.30 (1.52)	21.0
				FEV1/FVC	0.003	0.043	-0.09 (0.62)	0.0
	10- < 14	142 (16.3)	157 (38.3)	FEV1	0.149	0.315	-0.01 (1.11)	8.3
				FVC	0.137	0.291	-0.25 (0.94)	8.3
				FEV1/FVC	0.004	0.046	-0.08 (0.90)	5.1
	>=14	559 (64.3)	129 (31.5)	FEV1	0.192	0.349	-0.49 (1.04)	16.3
				FVC	0.186	0.347	-0.18 (0.79)	2.3
				FEV1/FVC	0.005	0.062	-0.25 (0.94)	4.7

Note: Age bands were defined as <10, 10 to <14, and>=14 years to quantify performance in younger children and adolescents. MSE and MAE are expressed on the original measurement scale; FEV1/FVC MSE and MAE use the 0–1 ratio scale. The study-model LLN used development-derived individualized sigma estimates

In the supplementary 5th-percentile quantile regression of FEV1/FVC z-scores (S1 and S2 Table), no equation showed significant residual association with age or height in either sex. In girls, age coefficients ranged from 0.017 to 0.047 z-score units per year across equations, and height coefficients ranged from -0.084 to 0.124 per 10 cm, all with p >= 0.280. In boys, age coefficients ranged from 0.021 to 0.104 per year, and height coefficients ranged from -0.276 to -0.154 per 10 cm, all with p >= 0.084. Thus, lower-tail FEV1/FVC calibration did not show statistically significant residual age- or height-related drift in the external-validation sample.

## Discussion

### Positioning the findings within pediatric spirometry reference-equation research

Accurate interpretation of spirometry depends on reference equations that capture the expected mean and dispersion of forced expiratory indices across growth. The GLI 2012 equations were developed to provide continuous, all-age reference values using large multinational datasets and modern distributional modeling. However, the GLI Task Force explicitly highlighted the need for local validation, particularly in populations that were underrepresented in the derivation samples [25]. In the Middle East, several groups have therefore developed population-specific equations, including a GAMLSS-based reference for Jordanian children aged 6–17 years. This reference was proposed as a regional option after showing age-dependent lack of fit of GLI 2012 in the same setting [11]. A large multicenter reference study has also been conducted in Saudi children and adolescents [12]. Similar motivations have driven updated national references in other regions with rapidly changing growth and environmental profiles, including Portugal [13] and China [14].

Within this context, the present study extends prior Middle Eastern efforts by directly comparing supervised predictive modeling strategies with established parametric reference-equation frameworks. A deliberate feature of the design was that phase 1 reused the derivation dataset from the original Jordanian GAMLSS study, enabling a controlled head-to-head comparison of modeling strategies under the same source data. The main finding was not that greater algorithmic complexity consistently improved prediction, but that appropriate transformation of age, height, and selected spirometric outcomes was central to model performance. Log-transformed specifications captured the nonlinear growth-related scaling of FEV1 and FVC, and simple transformed GLM models remained highly competitive with more flexible approaches. Where GBM models were selected, the gains were modest and depended on the same transformed predictor structure rather than on complex modeling alone. The independent phase 2 validation sample was therefore used to assess whether these functional-form choices and model-selection results transported beyond the derivation sample, because spirometry reference equations are often applied outside their development setting and calibration cannot be assumed.

### Positioning within digital health and real-world implementation

Contemporary digital health work increasingly emphasizes context-aware and user-facing systems rather than static equations alone. For example, Gavai and van Hillegersberg described a retrieval-augmented personalized-nutrition system for obesity and type 2 diabetes in which model outputs were connected to evidence sources, user characteristics, real-time recommendations, explainable feedback, and a prototype web application [18]. Similarly, Gavai and Meuwissen argued that digital-health ecosystems require coordination across multiple AI and data-sharing components, with explicit attention to governance, accountability, and interoperability [19]. These examples are not directly comparable to spirometry reference-equation modeling, but they illustrate the broader gap between prediction and deployment: a prediction model is only one layer of a digital-health system.

Within this broader context, the present study provides a candidate prediction and calibration layer for future pediatric spirometry software, rather than a complete digital-health intervention by itself. Its immediate contribution is to estimate expected spirometric values, individualized z-scores, and LLN thresholds in Jordanian children, and to compare these outputs with established equations. Future implementation would require embedding these models into spirometry software, electronic health records, or decision-support tools, where outputs could be linked to quality metrics, clinical context, prior lung-function trajectories, symptoms, asthma history, environmental exposures, and anthropometry. Interoperability standards such as Health Level Seven Fast Healthcare Interoperability Resources (HL7 FHIR) could support data exchange between spirometers, electronic health records, and clinical dashboards. However, technical integration alone would not establish clinical value; prospective evaluation would still be needed to assess workflow fit, user-interface clarity, clinician override behavior, calibration drift, subgroup performance, privacy safeguards, audit trails, and effects on referral or follow-up decisions.

### Interpretation of the superiority of log-log forms and its consistency with established modeling strategies

Across outcomes, performance was strongest when lung volumes were modeled using log-transformed predictors and responses. This finding is physiologically plausible because FEV1 and FVC scale allometrically with body size, and log-log specifications approximate multiplicative growth processes while reducing heteroscedasticity that otherwise increases at the extremes of age and height [6,26]. It is also consistent with the broader spirometry reference-equation literature, including the GLI 2012 approach, which used GAMLSS with transformations and smooth functions to capture nonlinear growth and changing variability across the life course [6].

Once this functional form was adopted, relatively simple models performed competitively with more flexible algorithms. This suggests that, in pediatric reference modeling based mainly on age and height, transformation and scale handling may be more important than added algorithmic complexity. Recent studies using flexible algorithms for spirometric prediction in large datasets have reported performance gains, but these gains depend on feature engineering and on how models account for scale and distributional structure [27,28]. From a translational perspective, log-log models also have a practical advantage: they remain interpretable, computationally simple, and feasible to implement in spirometry software, provided that predictions are back-transformed appropriately to the original clinical scale.

### Low explained variance of FEV1/FVC in context

FEV1/FVC remains clinically important because it is the main spirometric index used to identify airflow obstruction when the ratio falls below the lower limit of normal (LLN) under ERS/ATS interpretive standards [29]. In the present study, FEV1/FVC was also the outcome with the weakest explained variance showing very weak correlations across the study equation and comparator equations. This pattern is not unique to the present model or to the present dataset. Low explained variance for FEV1/FVC has been reported in other pediatric and reference-equation studies, even when FEV1 and FVC are predicted well. In a Navajo pediatric reference-equation study, demographic variables explained less than 5% of the variance in FEV1/FVC, while FEV1 and FVC were strongly related to height [30]. In the DARC birth cohort, height was the main predictor of spirometric indices in 6-year-old children except for FEV1/FVC [31]. The GLI 2012 report also showed that ethnic differences in FEV1 and FVC were largely proportional, leaving FEV1/FVC nearly independent of ethnic group [6]. This is biologically and mathematically plausible because FEV1/FVC is a ratio of two highly correlated lung volumes; growth-related variation that is strong in both FEV1 and FVC can be partly cancelled when the ratio is formed [32]. Residual variation in the ratio may instead reflect factors such as airway-lung size matching, dysanapsis, pubertal timing, adiposity, and measurement variability, which are not fully captured by chronological age and standing height [33,34]

The low R^2^ for FEV1/FVC should therefore be distinguished from the clinical role of the LLN. R^2^ describes how much variation in the continuous FEV1/FVC ratio is explained by predictors such as age and height. The LLN, in contrast, is a distributional cutoff intended to identify values in the lower tail of the healthy reference distribution, commonly corresponding to a z-score below -1.645 [6]. A model may explain little total variance in FEV1/FVC while still providing a clinically useful lower-tail threshold if the predicted mean and residual dispersion are adequately calibrated. For this reason, the diagnostic interpretation of obstruction should be based on whether FEV1/FVC falls below the LLN, not on the R^2^ value. In the present analysis, classification results based on FEV1/FVC were therefore interpreted through LLN-based calibration and below-LLN proportions, while acknowledging that age and height alone provide limited prediction of the continuous ratio.

### External validation relative to GLI and regional equations, and comparison with evidence from other populations

In external validation, the study model yielded a lower mean squared error for female FEV1 and FVC than GLI 2012, GLI 2022, and the Al-Qerem equation. For female FEV1/FVC, the study model performed slightly worse than the GLI equations. Among males, the study model did not improve FEV1 prediction, produced FVC error similar to GLI but higher than Al-Qerem, and had FEV1/FVC error similar to the comparator equations. These results indicate that model complexity did not drive performance. Simple GLM specifications remained competitive and were selected for both FVC models and for FEV1/FVC in girls, while GBM was selected for FEV1 in both sexes and for FEV1/FVC in boys. More flexible algorithms therefore did not consistently outperform simpler models or established reference-equation approaches. Similar conclusions have been reported in adult reference equation studies, where tree-based machine-learning models achieved performance comparable to distributional regression methods, with gains depending on the outcome and modeling choices.

The calibration shifts observed for the GLI and regional equations are consistent with the broader validation literature, where mean z-scores often deviate from 0 and LLN proportions depart from the expected 5% when equations are applied to new populations. For example, studies in Argentinian and South Italian children validating GLI 2012 reported systematic shifts in mean z-scores and variable miscalibration of LLN-based abnormality rates [35,36]. A population-based study of Swiss schoolchildren reported that GLI-based FEV1, FVC, and FEF25–75 z-scores were lower than expected, especially in adolescents, and specifically stratified children by age to assess systematic age-related deviations in GLI fit [37]. Pediatric studies comparing race-specific GLI 2012 equations with GLI Global or race-neutral equations have also shown that equation choice changes z-scores and interpretive classification in children, including healthy children in The Gambia and large clinical pediatric cohorts [38,39].

Although mean prediction error is useful for comparing equations, LLN-based interpretation depends on both the predicted mean and the residual dispersion used to generate z-scores. A reference equation can predict average spirometric values reasonably well but still classify too many or too few children below the LLN if the z-score distribution is not well calibrated in the target population [6,29]. This distinction is important for ventilatory-pattern classification, because obstruction depends on the FEV1/FVC LLN, whereas restrictive, nonspecific, and mixed patterns also depend on the FEV1 and FVC LLNs. Therefore, differences in below-LLN frequency and ventilatory-pattern classification in the present study should be interpreted primarily as evidence about equation calibration and transportability, rather than as estimates of disease prevalence. This interpretation is consistent with prior work showing that changing reference equations can materially alter spirometric classification in the same individuals [40,41].

The age-stratified findings should be interpreted as validation signals rather than as independent age-specific reference-equation derivations. The observed age-band differences in below-LLN frequency are based on subgroup proportions around the 5th-percentile threshold, and such proportions can fluctuate when sex-age strata are modest in size. This is relevant because the GLI sample-size analysis estimated that at least 150 males and 150 females are needed to validate published spirometry reference values with reasonable certainty, as smaller samples may produce apparent differences of up to approximately 0.5 z-scores from sampling error alone [42]. The present age-band results therefore identify where calibration departures appear in the external-validation sample, but they should not be read as definitive sex- and age-specific LLN frequencies.

Additional lower-tail analysis helps place this concern in context. The supplementary 5th-percentile quantile regression of FEV1/FVC z-scores found no statistically significant residual association with age or height in either sex across the study equation, Al-Qerem, GLI 2012, or GLI 2022. This is important because the analysis targeted the lower part of the z-score distribution most relevant to LLN-based obstruction classification, rather than average prediction alone. The absence of significant age- or height-related lower-tail drift reduces the likelihood that the FEV1/FVC LLN findings are simply an artifact of the derivation sample’s age structure.

The parallel pattern across equations is also informative. If the age structure of the derivation dataset were the sole explanation, departures would be expected to appear mainly in the study equation. Instead, broadly similar age-band behavior was observed across the study equation, Al-Qerem, GLI 2012, and GLI 2022, particularly for FEV1 and FVC in younger boys. This suggests a wider transportability issue related to growth stage, cohort structure, body proportions, secular trends, environmental exposures, or measurement context rather than a problem unique to one modeling strategy [37,43]. The findings therefore support cautious interpretation: age-band validation is useful for identifying calibration patterns, while the FEV1/FVC quantile regression suggests that LLN-relevant lower-tail behavior was not systematically driven by age or height in the validation sample.

### Strengths and limitations

An important strength of this study is its deliberate two-phase design. In the first phase, the same 1,576-child derivation dataset used for the original Jordanian GAMLSS reference equation was reused for the present predictive models. This produced a cleaner head-to-head comparison because the data source, population structure, and measurement context were held constant across the compared derivation methods. In the second phase, the models were tested in an independent 1,007-child validation dataset, allowing performance and calibration to be assessed outside the shared derivation sample. The 2025 external-validation sample was also temporally separated from the derivation dataset used for the 2021 regional equation, so the validation was not merely a same-period random split but a later, independently recruited transportability assessment.

Several additional strengths should be noted. The analysis compared simple transformed GLM specifications with more flexible predictive algorithms using the same sex-specific outcomes, predictor set, and resampling framework, which allowed model complexity to be evaluated directly rather than assumed to be beneficial. Candidate-model performance was calculated on the original clinical measurement scale, including back-transformed out-of-fold predictions for log-outcome models, making the error metrics directly comparable across model specifications. The study also evaluated equations beyond mean prediction error by examining z-score calibration, LLN proportions, ventilatory-pattern classification, age-stratified performance, and lower-tail FEV1/FVC behavior. Finally, the inclusion of GLI 2012, GLI 2022, and the regional Al-Qerem equation as comparators placed the proposed models in a clinically interpretable reference-equation context rather than treating prediction error alone as the endpoint.

A limitation in the present study is that predictor availability was intentionally restricted to age and height. This facilitates implementation and aligns with standard spirometry reference equations, but it may omit important determinants of lung growth, including pubertal status, body composition, nutritional status, and environmental exposures. Adiposity, for example, has been associated with higher FVC and lower FEV1/FVC in children and adolescents. This limitation is most evident for FEV1/FVC, where age and height explained little individual-level variation; however, low explained variance for this ratio is also reported in pediatric reference-equation literature and was also observed when GLI and Al-Qerem predictions were evaluated in the present validation sample. The derivation sample was not uniformly distributed by age, with older children contributing more observations than younger children, which may influence the precision of residual-SD calibration across growth bands. However, the younger age range was represented in model development, as every single-year age band from 6 to 17 years was included, with at least 40 observations per single-year band overall and 70–87 observations per year from ages 6–11 years. The different age structure of the external-validation dataset is therefore also informative, because it tests transportability in a sample that does not simply reproduce the derivation cohort. Importantly, elevated below-LLN proportions for FEV1 and FVC in boys younger than 10 years were also observed with GLI 2012, GLI 2022, and Al-Qerem equations, reducing the likelihood that this age-band pattern is solely a consequence of the derivation age distribution of the study model. Nevertheless, the age-stratified analyses should be interpreted cautiously because several sex-by-age validation strata were smaller than the sample sizes recommended for independent validation of spirometry reference values. The male validation group younger than 10 years, for example, included 124 children, and further stratification reduces the precision of LLN proportions. Larger multicenter datasets, ideally approaching or exceeding 150 boys and 150 girls within clinically relevant age bands, are needed before stable age-specific LLN frequencies can be estimated with confidence [42].

Recruitment from sports centers is another potential source of selection bias. Children engaged in regular swimming, taekwondo, or other organized activities may have higher average fitness or lung volumes than less active children. Although the sample also included schools, public parks, and Quran memorization centers, this recruitment pathway could bias predicted lung volumes upward and should be considered when applying the equations to less active pediatric populations.

Future work should prioritize multicenter external validation across neighboring countries. Where feasible, incorporating measures of adiposity and other markers of body build may improve transportability and reduce residual bias. Finally, clinical impact studies in symptomatic cohorts are needed to quantify how adopting different equations alters diagnostic classification and downstream management decisions, particularly given ongoing debate about the equity implications of reference equation choice. Future digital-health work should also test whether embedding the equation into spirometry software or electronic-health-record-linked decision support improves interpretation, referral behavior, repeat-testing decisions, and calibration monitoring across real clinical settings.

## Conclusion

In summary, comparative predictive modeling selected a mixture of simple and flexible specifications for pediatric spirometry in Jordanian children. GLM specifications remained competitive and were selected for both FVC models and for FEV1/FVC in girls, whereas GBM was selected for FEV1 in both sexes and for FEV1/FVC in boys. More complex machine-learning algorithms did not consistently outperform these simpler forms, indicating that functional form, scale handling, and calibration were more important than algorithmic complexity in this setting. Until studies in symptomatic patients and further refinement of distributional properties are available, clinicians and researchers should interpret differences in abnormality prevalence across equations as evidence of transportability and calibration differences rather than definitive evidence that one approach is clinically superior.

## Supporting information

S1 Table Lower-tail quantile regression of FEV1/FVC z-scores on age and height. (DOCX)

S2 Table Final model specifications, coefficients, hyperparameters, and residual-SD models. (DOCX)

S3 Table Code. Reproducible R implementation for pediatric spirometry predictive modeling. (DOCX)
