# Supplementary material for: Comparative predictive modeling of pediatric spirometry reference equations in Jordanian children: Complex versus simple models
Source: PLOS Digit Health. 2026 Sep 25;5(9):e0001746. doi: 10.1371/journal.pdig.0001746 (PMC13614654; doi:10.1371/journal.pdig.0001746)
Supplement: Supplementary file 1 [file pdig.0001746.s001.docx]

S1 Table. Lower-tail quantile regression of FEV1/FVC z-scores on age and height

| **Sex** | **Equation** | **Observed 5th percentile z** | **Age beta** | **Age p** | **Height beta per 10 cm** | **Height p** |
| --- | --- | --- | --- | --- | --- | --- |
| Female | Study predictive model | -1.727 | -0.057 | 0.308 | 0.124 | 0.370 |
|  | Al-Qerem | -1.704 | 0.047 | 0.280 | -0.084 | 0.439 |
|  | GLI 2012 Caucasian | -1.562 | 0.023 | 0.602 | -0.061 | 0.564 |
|  | GLI 2022 | -1.589 | 0.017 | 0.698 | -0.055 | 0.608 |
| Male | Study predictive model | -1.493 | 0.040 | 0.524 | -0.205 | 0.145 |
|  | Al-Qerem | -1.525 | 0.021 | 0.727 | -0.154 | 0.209 |
|  | GLI 2012 Caucasian | -1.496 | 0.104 | 0.171 | -0.276 | 0.084 |
|  | GLI 2022 | -1.531 | 0.079 | 0.310 | -0.227 | 0.163 |

Note. Z-scores were analyzed in the external-validation sample. The model was FEV1/FVC z-score ~ age_years + height_cm/10, fitted separately by sex and equation. Yellow shading indicates p < 0.05. FEV1 = forced expiratory volume in 1 second; FVC = forced vital capacity; GLI = Global Lung Function Initiative; LLN = lower limit of normal. Tau = 0.05 quantile regression was used because the lower limit of normal is a lower-tail cutoff. Coefficients represent conditional change in the 5th percentile of the FEV1/FVC z-score distribution per 1 year of age and per 10 cm of height, adjusted for the other predictor.
