# Supplementary material for: Comparative predictive modeling of pediatric spirometry reference equations in Jordanian children: Complex versus simple models
Source: PLOS Digit Health. 2026 Sep 25;5(9):e0001746. doi: 10.1371/journal.pdig.0001746 (PMC13614654; doi:10.1371/journal.pdig.0001746)
Supplement: Supplementary file 2 [file pdig.0001746.s002.docx]

# S2 Table. Final model specifications, coefficients, hyperparameters, and residual-SD models

This supplementary table provides the specifications needed to reproduce the final study equations. GLM components include coefficient estimates. GBM components are ensemble models and therefore do not have a single closed-form coefficient vector; reproducibility for GBM components is provided through fixed hyperparameters, and the prediction code in S1 Code.

## A. Final mean-prediction model specifications

| **Sex** | **Outcome** | **Model_class** | **Formula** | **Term** | **Estimate** | **Hyperparameters_or_details** | **Implementation_note** |
| --- | --- | --- | --- | --- | --- | --- | --- |
| Female | FEV1 | GBM | log(FEV1_L) ~ log(age_years) + log(height_cm) |  |  | n.trees=50; interaction.depth=2; shrinkage=0.1; n.minobsinnode=10 | GBM components require the fitted model object and prediction code; GLM coefficients are supplied directly. |
| Female | FVC | GLM Gaussian | FVC_L ~ age_years + height_cm | (Intercept) | -3.5077646471921 | none | GBM components require the fitted model object and prediction code; GLM coefficients are supplied directly. |
| Female | FVC | GLM Gaussian | FVC_L ~ age_years + height_cm | age_years | 0.0777032835036557 | none | GBM components require the fitted model object and prediction code; GLM coefficients are supplied directly. |
| Female | FVC | GLM Gaussian | FVC_L ~ age_years + height_cm | height_cm | 0.0356938579138624 | none | GBM components require the fitted model object and prediction code; GLM coefficients are supplied directly. |
| Female | FEV1/FVC | GLM Gaussian | FEV1FVC_ratio ~ log(age_years) + log(height_cm) | (Intercept) | 1.69911630900866 | none | GBM components require the fitted model object and prediction code; GLM coefficients are supplied directly. |
| Female | FEV1/FVC | GLM Gaussian | FEV1FVC_ratio ~ log(age_years) + log(height_cm) | log_age | 0.0171661241667394 | none | GBM components require the fitted model object and prediction code; GLM coefficients are supplied directly. |
| Female | FEV1/FVC | GLM Gaussian | FEV1FVC_ratio ~ log(age_years) + log(height_cm) | log_height | -0.168031001164838 | none | GBM components require the fitted model object and prediction code; GLM coefficients are supplied directly. |
| Male | FEV1 | GBM | log(FEV1_L) ~ log(age_years) + log(height_cm) |  |  | n.trees=150; interaction.depth=1; shrinkage=0.1; n.minobsinnode=10 | GBM components require the fitted model object and prediction code; GLM coefficients are supplied directly. |
| Male | FVC | GLM Gaussian | log(FVC_L) ~ log(age_years) + log(height_cm) | (Intercept) | -12.5749651978455 | none | GBM components require the fitted model object and prediction code; GLM coefficients are supplied directly. |
| Male | FVC | GLM Gaussian | log(FVC_L) ~ log(age_years) + log(height_cm) | log_age | 0.254988640049929 | none | GBM components require the fitted model object and prediction code; GLM coefficients are supplied directly. |
| Male | FVC | GLM Gaussian | log(FVC_L) ~ log(age_years) + log(height_cm) | log_height | 2.60019541576511 | none | GBM components require the fitted model object and prediction code; GLM coefficients are supplied directly. |
| Male | FEV1/FVC | GBM | FEV1FVC_ratio ~ log(age_years) + log(height_cm) |  |  | n.trees=50; interaction.depth=1; shrinkage=0.1; n.minobsinnode=10 | GBM components require the fitted model object and prediction code; GLM coefficients are supplied directly. |

## B. Development-derived individualized residual-SD model specifications

| **Sex** | **Outcome** | **Model_class** | **Formula** | **Prediction_scale** | **Hyperparameters_or_details** | **Implementation_note** |
| --- | --- | --- | --- | --- | --- | --- |
| Female | FEV1 | GBM | sigma_target ~ log_age + log_height | log outcome | Development OOF residual mean=0.000553757872757269; Development OOF residual SD=0.121642893683618; Sigma floor=0.059483746258981 | Residual SD was estimated from derivation-data out-of-fold residuals only; validation data were not used to estimate sigma. |
| Female | FVC | GLM | sigma_target ~ age_years + height_cm | original scale | Development OOF residual mean=0.000138824853874122; Development OOF residual SD=0.379809563273436; Sigma floor=0.213871753336307 | Residual SD was estimated from derivation-data out-of-fold residuals only; validation data were not used to estimate sigma. |
| Female | FEV1/FVC | GLM | sigma_target ~ log_age + log_height | original scale | Development OOF residual mean=-3.89127990560546e-05; Development OOF residual SD=0.0588018481472958; Sigma floor=0.0522294737261398 | Residual SD was estimated from derivation-data out-of-fold residuals only; validation data were not used to estimate sigma. |
| Male | FEV1 | GBM | sigma_target ~ log_age + log_height | log outcome | Development OOF residual mean=0.000250937691098445; Development OOF residual SD=0.125917224666157; Sigma floor=0.0984870275496989 | Residual SD was estimated from derivation-data out-of-fold residuals only; validation data were not used to estimate sigma. |
| Male | FVC | GLM | sigma_target ~ log_age + log_height | log outcome | Development OOF residual mean=2.76224499174438e-05; Development OOF residual SD=0.137421780612483; Sigma floor=0.121779625627838 | Residual SD was estimated from derivation-data out-of-fold residuals only; validation data were not used to estimate sigma. |
| Male | FEV1/FVC | GBM | sigma_target ~ log_age + log_height | original scale | Development OOF residual mean=-0.00067179141939711; Development OOF residual SD=0.0723776182320337; Sigma floor=0.0634165309979884 | Residual SD was estimated from derivation-data out-of-fold residuals only; validation data were not used to estimate sigma. |

## C. z-score and LLN calculation

| **Sex** | **Outcome** | **Model_class** | **Formula** | **Prediction_scale** | **Hyperparameters_or_details** | **Implementation_note** |
| --- | --- | --- | --- | --- | --- | --- |
| All | All | Normal-tail personalized-SD | LLN = predicted mean - 1.645 * individualized residual SD; z = (observed - predicted mean) / individualized residual SD | original scale for raw outcomes; log scale for log-outcome models followed by back-transformation | qnorm(0.05) = -1.64485362695147 | For log-outcome models, z-scores and LLNs are computed on the log scale and then back-transformed to the clinical scale. |

Abbreviations: FEV1 = forced expiratory volume in 1 second; FVC = forced vital capacity; FEV1/FVC = FEV1-to-FVC ratio on the 0-1 scale; GBM = gradient boosting machine; GLM = generalized linear model; LLN = lower limit of normal; SD = standard deviation.
