# Supplementary material for: Comparative predictive modeling of pediatric spirometry reference equations in Jordanian children: Complex versus simple models
Source: PLOS Digit Health. 2026 Sep 25;5(9):e0001746. doi: 10.1371/journal.pdig.0001746 (PMC13614654; doi:10.1371/journal.pdig.0001746)
Supplement: Supplementary file 3 [file pdig.0001746.s003.docx]

# S3 Code. Reproducible R implementation for pediatric spirometry predictive modeling

This supplementary file contains the final analysis code used for candidate-model comparison, final mean-model fitting, development-derived residual-SD modeling, z-score and LLN calculation, external validation, age-stratified validation, ventilatory-pattern classification, and the FEV1/FVC lower-tail quantile-regression analysis. The de-identified dataset is available from Zenodo as stated in the manuscript Data Availability Statement.

The code is presented in four sections. The first section is the master workflow. The second section generates original-scale candidate-model metrics. The third section fits final study models and development-derived sigma models. The fourth section performs the supplementary lower-tail quantile regression of FEV1/FVC z-scores.

## A. Master final workflow

```
# Supporting Information S2.
# Final reproducible R code for pediatric spirometry predictive modeling,
# external validation, z-score calculation, and LLN computation.
#
#

required_packages <- c("caret", "gbm", "randomForest", "kernlab", "ggplot2", "rspiro")
missing_packages <- required_packages[!vapply(required_packages, requireNamespace, logical(1), quietly = TRUE)]
if (length(missing_packages) > 0) {
  stop("Install required R packages before running this script: ", paste(missing_packages, collapse = ", "))
}

script_args <- commandArgs(trailingOnly = FALSE)
file_arg <- "--file="
script_path <- normalizePath(
  sub(file_arg, "", script_args[grep(file_arg, script_args)]),
  winslash = "/",
  mustWork = FALSE
)
script_dir <- if (length(script_path) == 0 || is.na(script_path)) getwd() else dirname(script_path)

required_files <- file.path(
  script_dir,
  c(
    "S1_deidentified_analysis_dataset_children.csv",
    "S2_candidate_model_original_scale_correction.R",
    "S2_downstream_development_sigma_lln.R"
  )
)
missing_files <- required_files[!file.exists(required_files)]
if (length(missing_files) > 0) {
  stop("Missing required file(s): ", paste(missing_files, collapse = "; "))
}

message("Running final candidate-model comparison...")
source(file.path(script_dir, "S2_candidate_model_original_scale_correction.R"), local = new.env(parent = globalenv()))

message("Running final development-data sigma LLN workflow...")
source(file.path(script_dir, "S2_downstream_development_sigma_lln.R"), local = new.env(parent = globalenv()))

expected_outputs <- file.path(
  script_dir,
  c(
    "candidate_model_performance_original_scale_all.csv",
    "Table2_candidate_models_boys_original_scale.csv",
    "Table3_candidate_models_girls_original_scale.csv",
    "S2_validation_predictions_development_sigma_lln.csv",
    "S2_development_sigma_model_report.csv",
    "S4_fitted_reference_models_development_sigma_lln.rds",
    "Table4_external_validation_development_sigma_lln.csv",
    "Table5_age_stratified_validation_development_sigma_lln.csv",
    "age_stratified_validation_by_equation_development_sigma_lln.csv",
    "Figure1_age_distribution_by_phase_sex.png",
    "Figure2_residuals_boys_development_sigma.png",
    "Figure3_residuals_girls_development_sigma.png",
    "below_LLN_by_equation_development_sigma_lln.csv",
    "ventilatory_patterns_development_sigma_lln.csv"
  )
)
missing_outputs <- expected_outputs[!file.exists(expected_outputs)]
if (length(missing_outputs) > 0) {
  stop("Final workflow did not generate expected output(s): ", paste(missing_outputs, collapse = "; "))
}

candidate_results <- read.csv(
  file.path(script_dir, "candidate_model_performance_original_scale_all.csv"),
  stringsAsFactors = FALSE
)
if (any(candidate_results$Metric_scale != "Original clinical measurement scale")) {
  stop("At least one candidate-model row is not on the original clinical measurement scale.")
}
ratio_candidate <- subset(candidate_results, Outcome == "FEV1/FVC")
if (nrow(ratio_candidate) == 0 || max(ratio_candidate$RMSE, ratio_candidate$MAE, na.rm = TRUE) > 0.2) {
  stop("FEV1/FVC candidate-model errors are not plausible ratio-scale values.")
}
if (any(!is.finite(candidate_results$R2) | candidate_results$R2 < 0 | candidate_results$R2 > 1)) {
  stop("Candidate-model R2 values must be finite squared correlations between 0 and 1.")
}

table4 <- read.csv(file.path(script_dir, "Table4_external_validation_development_sigma_lln.csv"), stringsAsFactors = FALSE)
if (!"R2" %in% names(table4)) {
  stop("Table 4 must include observed-predicted R2 for FEV1, FVC, and FEV1/FVC.")
}
if (any(!is.finite(table4$R2) | table4$R2 < 0 | table4$R2 > 1)) {
  stop("External-validation R2 values must be finite squared correlations between 0 and 1.")
}

ratio_predictions <- read.csv(
  file.path(script_dir, "S2_validation_predictions_development_sigma_lln.csv"),
  stringsAsFactors = FALSE
)
ratio_columns <- c("FEV1FVC_ratio", "pred_FEV1FVC_ratio", "LLN_FEV1FVC_ratio")
missing_ratio_columns <- ratio_columns[!ratio_columns %in% names(ratio_predictions)]
if (length(missing_ratio_columns) > 0) {
  stop("Missing ratio-scale FEV1/FVC column(s): ", paste(missing_ratio_columns, collapse = ", "))
}
if (max(unlist(ratio_predictions[, ratio_columns]), na.rm = TRUE) > 1.5) {
  stop("FEV1/FVC observed, predicted, and LLN values must be on the 0-1 ratio scale.")
}

session_info_path <- file.path(script_dir, "S2_R_session_info_final.txt")
writeLines(capture.output(sessionInfo()), session_info_path)

message("Final S2 workflow completed successfully.")
message("Session information written to: ", session_info_path)
```

## B. Candidate-model comparison on original clinical scale

```
# Corrected candidate-model comparison for pediatric spirometry reference equations.
#

required_packages <- c("caret", "gbm", "randomForest", "kernlab")
missing_packages <- required_packages[!vapply(required_packages, requireNamespace, logical(1), quietly = TRUE)]
if (length(missing_packages) > 0) {
  stop("Install required R packages before running this script: ", paste(missing_packages, collapse = ", "))
}

suppressPackageStartupMessages({
  library(caret)
  library(gbm)
  library(randomForest)
  library(kernlab)
})

set.seed(20260227)

script_args <- commandArgs(trailingOnly = FALSE)
file_arg <- "--file="
script_path <- normalizePath(
  sub(file_arg, "", script_args[grep(file_arg, script_args)]),
  winslash = "/",
  mustWork = FALSE
)
script_dir <- if (length(script_path) == 0 || is.na(script_path)) getwd() else dirname(script_path)
script_file_for_checks <- if (length(script_path) == 0 || is.na(script_path) || !file.exists(script_path)) {
  file.path(script_dir, "S2_candidate_model_original_scale_correction.R")
} else {
  script_path
}

input_path <- file.path(script_dir, "S1_deidentified_analysis_dataset_children.csv")
if (!file.exists(input_path)) {
  stop("Missing input file: ", input_path)
}

dat <- read.csv(input_path, stringsAsFactors = FALSE)

dat <- subset(
  dat,
  analysis_split == "training" &
    is.finite(age_years) & age_years > 0 &
    is.finite(height_cm) & height_cm > 0 &
    is.finite(FEV1_L) & FEV1_L > 0 &
    is.finite(FVC_L) & FVC_L > 0
)

if ("FEV1FVC_ratio" %in% names(dat)) {
  dat$FEV1FVC_ratio_model <- as.numeric(dat$FEV1FVC_ratio)
  fev1fvc_storage_note <- "Raw dataset includes FEV1FVC_ratio; this ratio-scale variable was used for candidate models."
} else if ("FEV1FVC_percent" %in% names(dat)) {
  dat$FEV1FVC_ratio_model <- as.numeric(dat$FEV1FVC_percent) / 100
  fev1fvc_storage_note <- "Raw dataset stored FEV1/FVC as percentage only; candidate models used FEV1FVC_percent / 100."
} else {
  stop("Missing FEV1/FVC variable. Expected FEV1FVC_ratio or FEV1FVC_percent.")
}

dat <- subset(dat, is.finite(FEV1FVC_ratio_model) & FEV1FVC_ratio_model > 0)
if (max(dat$FEV1FVC_ratio_model, na.rm = TRUE) > 1.5) {
  stop("FEV1/FVC values are not on a plausible ratio scale after standardization.")
}

dat$sex <- factor(dat$sex, levels = c("Female", "Male"))
dat$log_age <- log(dat$age_years)
dat$log_height <- log(dat$height_cm)

outcome_specs <- list(
  FEV1 = list(var = "FEV1_L", display = "FEV1", unit = "L"),
  FVC = list(var = "FVC_L", display = "FVC", unit = "L"),
  FEV1FVC = list(var = "FEV1FVC_ratio_model", display = "FEV1/FVC", unit = "ratio")
)

framework_specs <- list(
  raw_raw = list(
    label = "Raw predictors -> raw outcome",
    predictors = c("age_years", "height_cm"),
    outcome_scale = "raw"
  ),
  logpred_raw = list(
    label = "Log(age,height) -> raw outcome",
    predictors = c("log_age", "log_height"),
    outcome_scale = "raw"
  ),
  logpred_logout = list(
    label = "Log(age,height) -> log(outcome)",
    predictors = c("log_age", "log_height"),
    outcome_scale = "log"
  )
)

algorithm_specs <- list(
  GLM = list(method = "glm", tuneLength = 1),
  GBM = list(method = "gbm", tuneLength = 3),
  RF = list(method = "rf", tuneLength = 3),
  KNN = list(method = "knn", tuneLength = 3),
  SVM = list(method = "svmRadial", tuneLength = 3)
)

correlation_r2 <- function(obs, pred) {
  keep <- is.finite(obs) & is.finite(pred)
  if (sum(keep) < 2) {
    return(NA_real_)
  }
  stats::cor(obs[keep], pred[keep])^2
}

metric_summary <- function(obs, pred) {
  data.frame(
    RMSE = sqrt(mean((obs - pred)^2, na.rm = TRUE)),
    MAE = mean(abs(obs - pred), na.rm = TRUE),
    MSE = mean((obs - pred)^2, na.rm = TRUE),
    R2 = correlation_r2(obs, pred),
    N = sum(is.finite(obs) & is.finite(pred))
  )
}

format_tune <- function(df) {
  tune_cols <- setdiff(names(df), c("pred", "obs", "rowIndex", "Resample"))
  if (length(tune_cols) == 0) {
    return(rep("none", nrow(df)))
  }
  apply(df[, tune_cols, drop = FALSE], 1, function(x) {
    paste(paste(names(x), x, sep = "="), collapse = "; ")
  })
}

make_formula <- function(predictors) {
  as.formula(paste("target ~", paste(predictors, collapse = " + ")))
}

run_one_candidate <- function(data_sex, sex_label, outcome_name, framework_name, algorithm_name, ctrl) {
  outcome_spec <- outcome_specs[[outcome_name]]
  framework <- framework_specs[[framework_name]]
  algorithm <- algorithm_specs[[algorithm_name]]

  model_data <- data_sex
  model_data$observed_original <- as.numeric(model_data[[outcome_spec$var]])
  if (framework$outcome_scale == "log") {
    model_data$target <- log(model_data$observed_original)
  } else {
    model_data$target <- model_data$observed_original
  }

  model_data <- model_data[complete.cases(model_data[, c("target", "observed_original", framework$predictors)]), ]

  train_args <- list(
    form = make_formula(framework$predictors),
    data = model_data,
    method = algorithm$method,
    trControl = ctrl,
    tuneLength = algorithm$tuneLength,
    metric = "RMSE"
  )
  if (algorithm_name == "GBM") {
    train_args$verbose <- FALSE
  }
  fit <- do.call(train, train_args)

  pred_df <- fit$pred
  if (!nrow(pred_df)) {
    stop("No out-of-fold predictions were saved for ", sex_label, " ", outcome_name, " ", framework$label, " ", algorithm_name)
  }

  pred_df$tune_id <- format_tune(pred_df)
  pred_df$observed_original <- model_data$observed_original[pred_df$rowIndex]

  if (framework$outcome_scale == "log") {
    pred_df$pred_original <- exp(pred_df$pred)
    back_transform <- "Direct exponential back-transformation of each out-of-fold predicted value; no smearing correction."
  } else {
    pred_df$pred_original <- pred_df$pred
    back_transform <- "No back-transformation required."
  }

  split_pred <- split(pred_df, pred_df$tune_id)
  metrics <- do.call(
    rbind,
    lapply(names(split_pred), function(tune_id) {
      d <- split_pred[[tune_id]]
      out <- metric_summary(d$observed_original, d$pred_original)
      out$tune_id <- tune_id
      out
    })
  )
  rownames(metrics) <- NULL
  best <- metrics[order(metrics$RMSE, metrics$MAE), ][1, ]

  data.frame(
    Sex = sex_label,
    Outcome = outcome_spec$display,
    Framework = framework$label,
    Outcome_fitted_scale = framework$outcome_scale,
    Algorithm = algorithm_name,
    Tune = best$tune_id,
    RMSE = best$RMSE,
    MAE = best$MAE,
    MSE = best$MSE,
    R2 = best$R2,
    N = best$N,
    Metric_scale = "Original clinical measurement scale",
    Back_transform = back_transform,
    stringsAsFactors = FALSE
  )
}

all_results <- list()

for (sex_label in c("Female", "Male")) {
  data_sex <- subset(dat, sex == sex_label)
  set.seed(20260227 + ifelse(sex_label == "Female", 1, 2))
  folds <- createFolds(seq_len(nrow(data_sex)), k = 5, returnTrain = TRUE)
  ctrl <- trainControl(
    method = "cv",
    number = 5,
    index = folds,
    savePredictions = "all",
    allowParallel = FALSE
  )

  for (outcome_name in names(outcome_specs)) {
    for (framework_name in names(framework_specs)) {
      for (algorithm_name in names(algorithm_specs)) {
        message("Running ", sex_label, " / ", outcome_name, " / ", framework_specs[[framework_name]]$label, " / ", algorithm_name)
        all_results[[length(all_results) + 1]] <- run_one_candidate(
          data_sex = data_sex,
          sex_label = sex_label,
          outcome_name = outcome_name,
          framework_name = framework_name,
          algorithm_name = algorithm_name,
          ctrl = ctrl
        )
      }
    }
  }
}

results <- do.call(rbind, all_results)

# Safety checks against the previous scale error.
script_text <- if (file.exists(script_file_for_checks)) {
  paste(readLines(script_file_for_checks, warn = FALSE), collapse = "\n")
} else {
  ""
}
bad_exp_pattern <- paste0("exp", "\\s*", "\\(", "\\s*", "(RMSE|MAE|MSE)")
if (grepl(bad_exp_pattern, script_text, ignore.case = TRUE)) {
  stop("Invalid exponentiation of summary error metrics detected.")
}
if (any(results$Metric_scale != "Original clinical measurement scale")) {
  stop("At least one candidate row is not marked as original-scale.")
}
ratio_rows <- results$Outcome == "FEV1/FVC"
if (any(results$RMSE[ratio_rows] > 0.20 | results$MAE[ratio_rows] > 0.20, na.rm = TRUE)) {
  stop("FEV1/FVC candidate errors are too large for ratio-scale metrics; check scale handling.")
}

write.csv(
  results,
  file.path(script_dir, "candidate_model_performance_original_scale_all_unselected.csv"),
  row.names = FALSE
)

results$Selected_within_sex_outcome <- FALSE
for (group_id in unique(interaction(results$Sex, results$Outcome, drop = TRUE))) {
  idx <- interaction(results$Sex, results$Outcome, drop = TRUE) == group_id
  best_idx <- which(idx)[which.min(results$RMSE[idx])]
  results$Selected_within_sex_outcome[best_idx] <- TRUE
}

results <- results[order(results$Sex, match(results$Outcome, c("FEV1", "FVC", "FEV1/FVC")), results$RMSE), ]
write.csv(
  results,
  file.path(script_dir, "candidate_model_performance_original_scale_all.csv"),
  row.names = FALSE
)

table_for_manuscript <- function(sex_label) {
  out <- subset(results, Sex == sex_label)
  out <- out[order(match(out$Outcome, c("FEV1", "FVC", "FEV1/FVC")), out$Framework, out$Algorithm), ]
  out$RMSE <- round(out$RMSE, 3)
  out$MAE <- round(out$MAE, 3)
  out$MSE <- round(out$MSE, 3)
  out$R2 <- round(out$R2, 3)
  out[, c("Outcome", "Framework", "Algorithm", "RMSE", "MAE", "MSE", "R2", "Selected_within_sex_outcome")]
}

table3_girls <- table_for_manuscript("Female")
table2_boys <- table_for_manuscript("Male")

write.csv(table2_boys, file.path(script_dir, "Table2_candidate_models_boys_original_scale.csv"), row.names = FALSE)
write.csv(table3_girls, file.path(script_dir, "Table3_candidate_models_girls_original_scale.csv"), row.names = FALSE)

current_final <- data.frame(
  Sex = c("Female", "Female", "Female", "Male", "Male", "Male"),
  Outcome = c("FEV1", "FVC", "FEV1/FVC", "FEV1", "FVC", "FEV1/FVC"),
  Current_Framework = c(
    "Log(age,height) -> log(outcome)",
    "Log(age,height) -> log(outcome)",
    "Log(age,height) -> raw outcome",
    "Log(age,height) -> log(outcome)",
    "Log(age,height) -> log(outcome)",
    "Log(age,height) -> raw outcome"
  ),
  Current_Algorithm = c("GBM", "GLM", "GBM", "GLM", "GLM", "GBM"),
  stringsAsFactors = FALSE
)

selected <- subset(results, Selected_within_sex_outcome)
comparison <- merge(
  current_final,
  selected[, c("Sex", "Outcome", "Framework", "Algorithm", "RMSE", "MAE", "MSE", "R2")],
  by = c("Sex", "Outcome"),
  all.x = TRUE
)
comparison$Selected_changed <- with(
  comparison,
  Framework != Current_Framework | Algorithm != Current_Algorithm
)
comparison <- comparison[order(comparison$Sex, match(comparison$Outcome, c("FEV1", "FVC", "FEV1/FVC"))), ]
write.csv(comparison, file.path(script_dir, "candidate_model_selection_comparison_original_scale.csv"), row.names = FALSE)

report <- c(
  "Corrected candidate-model comparison on original clinical scale",
  "",
  paste0("Input dataset: ", input_path),
  paste0("Training rows analyzed: ", nrow(dat)),
  fev1fvc_storage_note,
  "FEV1 and FVC were analyzed in liters.",
  "FEV1/FVC was standardized to the 0-1 ratio scale before fitting any candidate model.",
  "For log-outcome models, out-of-fold predicted log values were back-transformed at the individual level using exp(predicted_log_value).",
  "No smearing correction was used; direct exponential back-transformation was used for the candidate-model comparison.",
  "RMSE, MAE, MSE, and squared-correlation R2 were calculated after back-transformation on the original clinical measurement scale.",
  "R2 definition: cor(observed, predicted)^2, calculated from out-of-fold predictions on the original clinical measurement scale.",
  "",
  "Selected model comparison:",
  paste(capture.output(print(comparison, row.names = FALSE)), collapse = "\n"),
  "",
  if (any(comparison$Selected_changed, na.rm = TRUE)) {
    "At least one selected final model changed after original-scale correction; downstream workflow must be rerun for affected combinations."
  } else {
    "No selected final models changed after original-scale correction."
  }
)
writeLines(report, file.path(script_dir, "candidate_model_original_scale_analysis_report.txt"))

message("Completed corrected candidate-model comparison. Outputs written to: ", script_dir)
```

## C. Final development-data sigma LLN workflow

```
# Downstream workflow using development-data sigma models for LLN estimation.

required_packages <- c("caret", "gbm", "ggplot2", "rspiro")
missing_packages <- required_packages[!vapply(required_packages, requireNamespace, logical(1), quietly = TRUE)]
if (length(missing_packages) > 0) {
  stop("Install required R packages before running this script: ", paste(missing_packages, collapse = ", "))
}

suppressPackageStartupMessages({
  library(caret)
  library(gbm)
  library(ggplot2)
  library(rspiro)
})

set.seed(20260227)

script_args <- commandArgs(trailingOnly = FALSE)
file_arg <- "--file="
script_path <- normalizePath(
  sub(file_arg, "", script_args[grep(file_arg, script_args)]),
  winslash = "/",
  mustWork = FALSE
)
script_dir <- if (length(script_path) == 0 || is.na(script_path)) getwd() else dirname(script_path)

input_path <- file.path(script_dir, "S1_deidentified_analysis_dataset_children.csv")
dat <- read.csv(input_path, stringsAsFactors = FALSE)

dat <- subset(
  dat,
  is.finite(age_years) & age_years > 0 &
    is.finite(height_cm) & height_cm > 0 &
    is.finite(FEV1_L) & FEV1_L > 0 &
    is.finite(FVC_L) & FVC_L > 0
)

if ("FEV1FVC_ratio" %in% names(dat)) {
  dat$FEV1FVC_ratio_model <- as.numeric(dat$FEV1FVC_ratio)
} else if ("FEV1FVC_percent" %in% names(dat)) {
  dat$FEV1FVC_ratio_model <- as.numeric(dat$FEV1FVC_percent) / 100
} else {
  stop("Missing FEV1/FVC variable. Expected FEV1FVC_ratio or FEV1FVC_percent.")
}

dat <- subset(dat, is.finite(FEV1FVC_ratio_model) & FEV1FVC_ratio_model > 0)
if (max(dat$FEV1FVC_ratio_model, na.rm = TRUE) > 1.5) {
  stop("FEV1/FVC values are not on a plausible ratio scale after standardization.")
}

dat$sex <- factor(dat$sex, levels = c("Female", "Male"))
dat$log_age <- log(dat$age_years)
dat$log_height <- log(dat$height_cm)
dat$log_FEV1 <- log(dat$FEV1_L)
dat$log_FVC <- log(dat$FVC_L)

gli_global_gender <- ifelse(dat$sex == "Male", 1, ifelse(dat$sex == "Female", 2, NA_real_))
gli_global_pred <- pred_GLIgl(
  age = dat$age_years,
  height = dat$height_cm / 100,
  gender = gli_global_gender,
  param = c("FEV1", "FVC", "FEV1FVC")
)
gli_global_z <- zscore_GLIgl(
  age = dat$age_years,
  height = dat$height_cm / 100,
  gender = gli_global_gender,
  FEV1 = dat$FEV1_L,
  FVC = dat$FVC_L,
  FEV1FVC = dat$FEV1FVC_ratio_model
)

dat$GLIgl_FEV1_pred <- gli_global_pred$pred.FEV1
dat$GLIgl_FVC_pred <- gli_global_pred$pred.FVC
dat$GLIgl_FEV1FVC_pred <- gli_global_pred$pred.FEV1FVC
dat$GLIgl_FEV1_z <- gli_global_z$z.score.FEV1
dat$GLIgl_FVC_z <- gli_global_z$z.score.FVC
dat$GLIgl_FEV1FVC_z <- gli_global_z$z.score.FEV1FVC

if (all(c("GLI2012_FEV1_pred", "GLI2012_FVC_pred", "GLI2012_FEV1FVC_pred") %in% names(dat))) {
  gli_difference_check <- max(
    abs(dat$GLIgl_FEV1_pred - dat$GLI2012_FEV1_pred),
    abs(dat$GLIgl_FVC_pred - dat$GLI2012_FVC_pred),
    abs(dat$GLIgl_FEV1FVC_pred - dat$GLI2012_FEV1FVC_pred),
    na.rm = TRUE
  )
  if (!is.finite(gli_difference_check) || gli_difference_check < 1e-6) {
    stop("Recomputed GLI 2022 predictions are unexpectedly identical to GLI 2012 predictions.")
  }
}

training <- subset(dat, analysis_split == "training")
validation <- subset(dat, analysis_split == "external_validation")

figure1_source <- subset(dat, analysis_split %in% c("training", "external_validation"))
figure1_source$Study_phase <- factor(
  ifelse(figure1_source$analysis_split == "training", "Training", "Validation"),
  levels = c("Training", "Validation")
)
figure1_age_bin_levels <- c("6-<8", "8-<10", "10-<12", "12-<14", "14-<16", "16-18")
figure1_source$Age_bin <- cut(
  figure1_source$age_years,
  breaks = c(6, 8, 10, 12, 14, 16, Inf),
  labels = figure1_age_bin_levels,
  right = FALSE,
  include.lowest = TRUE
)
figure1_rows <- list()
for (phase in levels(figure1_source$Study_phase)) {
  phase_data <- subset(figure1_source, Study_phase == phase)
  for (group in c("Female", "Male", "Total")) {
    group_data <- if (group == "Total") phase_data else subset(phase_data, sex == group)
    tab <- prop.table(table(factor(group_data$Age_bin, levels = levels(figure1_source$Age_bin)))) * 100
    figure1_rows[[length(figure1_rows) + 1]] <- data.frame(
      Study_phase = phase,
      Sex_group = group,
      Age_bin = names(tab),
      Percent = as.numeric(tab),
      stringsAsFactors = FALSE
    )
  }
}
figure1_df <- do.call(rbind, figure1_rows)
figure1_df$Sex_group <- factor(figure1_df$Sex_group, levels = c("Female", "Male", "Total"))
figure1_df$Age_bin <- factor(figure1_df$Age_bin, levels = figure1_age_bin_levels)
figure1_plot <- ggplot(figure1_df, aes(x = Age_bin, y = Percent, fill = Sex_group)) +
  geom_col(position = position_dodge(width = 0.85), width = 0.8) +
  facet_wrap(~Study_phase, nrow = 1) +
  scale_fill_manual(values = c("Female" = "#F8766D", "Male" = "#00BA38", "Total" = "#619CFF")) +
  labs(x = "Age bin (years)", y = "Percentage within phase and sex (%)", fill = "Sex group") +
  theme_bw(base_size = 11) +
  theme(axis.text.x = element_text(angle = 0, hjust = 0.5))
ggsave(file.path(script_dir, "Figure1_age_distribution_by_phase_sex.png"), figure1_plot, width = 7, height = 4.5, dpi = 300)

gbm_grid_female_fev1 <- expand.grid(n.trees = 50, interaction.depth = 2, shrinkage = 0.1, n.minobsinnode = 10)
gbm_grid_male_fev1 <- expand.grid(n.trees = 150, interaction.depth = 1, shrinkage = 0.1, n.minobsinnode = 10)
gbm_grid_male_ratio <- expand.grid(n.trees = 50, interaction.depth = 1, shrinkage = 0.1, n.minobsinnode = 10)

ctrl_none <- trainControl(method = "none")
q05 <- qnorm(0.05)
eps <- 1e-8
expected_log_chisq1 <- digamma(0.5) + log(2)

model_specs <- list(
  Female = list(
    FEV1 = list(
      outcome = "FEV1_L",
      label = "FEV1",
      formula = "log_FEV1 ~ log_age + log_height",
      sigma_formula = "sigma_target ~ log_age + log_height",
      method = "gbm",
      tuneGrid = gbm_grid_female_fev1,
      transform = "log",
      ratio = FALSE
    ),
    FVC = list(
      outcome = "FVC_L",
      label = "FVC",
      formula = "FVC_L ~ age_years + height_cm",
      sigma_formula = "sigma_target ~ age_years + height_cm",
      method = "glm",
      tuneGrid = NULL,
      transform = "raw",
      ratio = FALSE
    ),
    FEV1FVC = list(
      outcome = "FEV1FVC_ratio_model",
      label = "FEV1/FVC",
      formula = "FEV1FVC_ratio_model ~ log_age + log_height",
      sigma_formula = "sigma_target ~ log_age + log_height",
      method = "glm",
      tuneGrid = NULL,
      transform = "raw",
      ratio = TRUE
    )
  ),
  Male = list(
    FEV1 = list(
      outcome = "FEV1_L",
      label = "FEV1",
      formula = "log_FEV1 ~ log_age + log_height",
      sigma_formula = "sigma_target ~ log_age + log_height",
      method = "gbm",
      tuneGrid = gbm_grid_male_fev1,
      transform = "log",
      ratio = FALSE
    ),
    FVC = list(
      outcome = "FVC_L",
      label = "FVC",
      formula = "log_FVC ~ log_age + log_height",
      sigma_formula = "sigma_target ~ log_age + log_height",
      method = "glm",
      tuneGrid = NULL,
      transform = "log",
      ratio = FALSE
    ),
    FEV1FVC = list(
      outcome = "FEV1FVC_ratio_model",
      label = "FEV1/FVC",
      formula = "FEV1FVC_ratio_model ~ log_age + log_height",
      sigma_formula = "sigma_target ~ log_age + log_height",
      method = "gbm",
      tuneGrid = gbm_grid_male_ratio,
      transform = "raw",
      ratio = TRUE
    )
  )
)

fit_train_model <- function(formula_text, data, spec) {
  args <- list(
    form = as.formula(formula_text),
    data = data,
    method = spec$method,
    trControl = ctrl_none
  )
  if (!is.null(spec$tuneGrid)) {
    args$tuneGrid <- spec$tuneGrid
  }
  if (identical(spec$method, "gbm")) {
    args$verbose <- FALSE
  }
  do.call(train, args)
}

observed_analysis <- function(data, spec) {
  x <- data[[spec$outcome]]
  if (identical(spec$transform, "log")) {
    return(log(x))
  }
  x
}

clinical_from_analysis <- function(x, spec) {
  out <- if (identical(spec$transform, "log")) exp(x) else x
  if (isTRUE(spec$ratio)) {
    out <- pmin(pmax(out, 0), 1.5)
  }
  out
}

analysis_from_clinical <- function(data, spec) {
  observed_analysis(data, spec)
}

predict_analysis <- function(fit, data) {
  as.numeric(predict(fit, newdata = data))
}

make_oof_residuals <- function(train_sex, spec) {
  y <- observed_analysis(train_sex, spec)
  folds <- createFolds(y, k = 5, list = TRUE, returnTrain = FALSE)
  pred <- rep(NA_real_, nrow(train_sex))

  for (fold_id in seq_along(folds)) {
    holdout <- folds[[fold_id]]
    fit <- fit_train_model(spec$formula, train_sex[-holdout, , drop = FALSE], spec)
    pred[holdout] <- predict_analysis(fit, train_sex[holdout, , drop = FALSE])
  }

  y - pred
}

fit_sigma_model <- function(train_sex, spec, oof_resid) {
  bias <- mean(oof_resid, na.rm = TRUE)
  centered_resid <- oof_resid - bias
  sigma_train <- train_sex
  sigma_train$sigma_target <- log(centered_resid^2 + eps)

  fit <- fit_train_model(spec$sigma_formula, sigma_train, spec)
  dev_sigma <- pmax(sqrt(exp(predict_analysis(fit, sigma_train) - expected_log_chisq1)), sqrt(eps))

  list(
    model = fit,
    bias = bias,
    floor = unname(quantile(dev_sigma, 0.01, na.rm = TRUE)),
    sigma_scale = ifelse(identical(spec$transform, "log"), "log outcome", "original scale")
  )
}

fit_all_for_sex <- function(train_sex, sex_label) {
  specs <- model_specs[[sex_label]]
  mean_models <- list()
  sigma_models <- list()
  sigma_report <- list()

  for (outcome_name in names(specs)) {
    spec <- specs[[outcome_name]]
    mean_models[[outcome_name]] <- fit_train_model(spec$formula, train_sex, spec)
    oof_resid <- make_oof_residuals(train_sex, spec)
    sigma_models[[outcome_name]] <- fit_sigma_model(train_sex, spec, oof_resid)

    sigma_report[[length(sigma_report) + 1]] <- data.frame(
      Sex = sex_label,
      Outcome = spec$label,
      Model_method = toupper(spec$method),
      Mean_formula = spec$formula,
      Sigma_formula = spec$sigma_formula,
      Sigma_scale = sigma_models[[outcome_name]]$sigma_scale,
      Development_OOF_residual_mean = mean(oof_resid, na.rm = TRUE),
      Development_OOF_residual_SD = sd(oof_resid, na.rm = TRUE),
      Sigma_floor = sigma_models[[outcome_name]]$floor,
      stringsAsFactors = FALSE
    )
  }

  list(
    mean_models = mean_models,
    sigma_models = sigma_models,
    sigma_report = do.call(rbind, sigma_report)
  )
}

predict_reference_outcome <- function(data_sex, spec, mean_fit, sigma_fit) {
  pred_analysis_value <- predict_analysis(mean_fit, data_sex) + sigma_fit$bias
  sigma <- pmax(
    sqrt(exp(predict_analysis(sigma_fit$model, data_sex) - expected_log_chisq1)),
    sigma_fit$floor
  )
  obs_analysis <- analysis_from_clinical(data_sex, spec)
  z <- (obs_analysis - pred_analysis_value) / sigma
  lln_analysis <- pred_analysis_value + q05 * sigma

  list(
    pred = clinical_from_analysis(pred_analysis_value, spec),
    sigma = sigma,
    z = z,
    lln = clinical_from_analysis(lln_analysis, spec)
  )
}

predict_reference_for_sex <- function(data_sex, sex_label, fitted) {
  specs <- model_specs[[sex_label]]
  pred_fev1 <- predict_reference_outcome(data_sex, specs$FEV1, fitted$mean_models$FEV1, fitted$sigma_models$FEV1)
  pred_fvc <- predict_reference_outcome(data_sex, specs$FVC, fitted$mean_models$FVC, fitted$sigma_models$FVC)
  pred_ratio <- predict_reference_outcome(data_sex, specs$FEV1FVC, fitted$mean_models$FEV1FVC, fitted$sigma_models$FEV1FVC)

  data.frame(
    participant_id = data_sex$participant_id,
    sex = as.character(data_sex$sex),
    age_years = data_sex$age_years,
    height_cm = data_sex$height_cm,
    FEV1_L = data_sex$FEV1_L,
    FVC_L = data_sex$FVC_L,
    FEV1FVC_ratio = data_sex$FEV1FVC_ratio_model,
    pred_FEV1_L = pred_fev1$pred,
    sigma_FEV1 = pred_fev1$sigma,
    z_FEV1 = pred_fev1$z,
    LLN_FEV1_L = pred_fev1$lln,
    pred_FVC_L = pred_fvc$pred,
    sigma_FVC = pred_fvc$sigma,
    z_FVC = pred_fvc$z,
    LLN_FVC_L = pred_fvc$lln,
    pred_FEV1FVC_ratio = pred_ratio$pred,
    sigma_FEV1FVC = pred_ratio$sigma,
    z_FEV1FVC = pred_ratio$z,
    LLN_FEV1FVC_ratio = pred_ratio$lln
  )
}

female_fitted <- fit_all_for_sex(subset(training, sex == "Female"), "Female")
male_fitted <- fit_all_for_sex(subset(training, sex == "Male"), "Male")

saveRDS(
  list(
    mean_models = list(Female = female_fitted$mean_models, Male = male_fitted$mean_models),
    sigma_models = list(Female = female_fitted$sigma_models, Male = male_fitted$sigma_models),
    note = "Selected mean models with development-data sigma models for normal-tail LLN estimation."
  ),
  file.path(script_dir, "S4_fitted_reference_models_development_sigma_lln.rds")
)

pred <- rbind(
  predict_reference_for_sex(subset(validation, sex == "Female"), "Female", female_fitted),
  predict_reference_for_sex(subset(validation, sex == "Male"), "Male", male_fitted)
)

write.csv(pred, file.path(script_dir, "S2_validation_predictions_development_sigma_lln.csv"), row.names = FALSE)
write.csv(
  rbind(female_fitted$sigma_report, male_fitted$sigma_report),
  file.path(script_dir, "S2_development_sigma_model_report.csv"),
  row.names = FALSE
)

validation2 <- merge(validation, pred, by = c("participant_id", "sex", "age_years", "height_cm", "FEV1_L", "FVC_L"), all.x = TRUE)
validation2$FEV1FVC_ratio.x <- NULL

ratio_pred <- function(x) {
  x <- as.numeric(x)
  ifelse(is.finite(x) & abs(x) > 2, x / 100, x)
}

equations <- list(
  "Al-Qerem" = list(
    FEV1 = list(pred = "ALQ_FEV1_pred", z = "ALQ_FEV1_z"),
    FVC = list(pred = "ALQ_FVC_pred", z = "ALQ_FVC_z"),
    FEV1FVC = list(pred = "ALQ_FEV1FVC_pred", z = "ALQ_FEV1FVC_z", ratio_pred = TRUE)
  ),
  "GLI 2012 Caucasian" = list(
    FEV1 = list(pred = "GLI2012_FEV1_pred", z = "GLI2012_FEV1_z"),
    FVC = list(pred = "GLI2012_FVC_pred", z = "GLI2012_FVC_z"),
    FEV1FVC = list(pred = "GLI2012_FEV1FVC_pred", z = "GLI2012_FEV1FVC_z", ratio_pred = TRUE)
  ),
  "GLI 2022" = list(
    FEV1 = list(pred = "GLIgl_FEV1_pred", z = "GLIgl_FEV1_z"),
    FVC = list(pred = "GLIgl_FVC_pred", z = "GLIgl_FVC_z"),
    FEV1FVC = list(pred = "GLIgl_FEV1FVC_pred", z = "GLIgl_FEV1FVC_z", ratio_pred = TRUE)
  ),
  "Study predictive model" = list(
    FEV1 = list(pred = "pred_FEV1_L", z = "z_FEV1"),
    FVC = list(pred = "pred_FVC_L", z = "z_FVC"),
    FEV1FVC = list(pred = "pred_FEV1FVC_ratio", z = "z_FEV1FVC", ratio_pred = TRUE)
  )
)

observed_column <- function(outcome) {
  if (outcome == "FEV1") return("FEV1_L")
  if (outcome == "FVC") return("FVC_L")
  "FEV1FVC_ratio_model"
}

mse_ci <- function(err2) {
  m <- mean(err2, na.rm = TRUE)
  se <- sd(err2, na.rm = TRUE) / sqrt(sum(is.finite(err2)))
  c(m, max(0, m - 1.96 * se), m + 1.96 * se)
}

z_cell <- function(z) {
  z <- z[is.finite(z)]
  mean_p <- tryCatch(t.test(z, mu = 0)$p.value, error = function(e) NA_real_)
  sd_z <- sd(z)
  n <- length(z)
  chi <- (n - 1) * var(z)
  var_p <- 2 * min(pchisq(chi, df = n - 1), 1 - pchisq(chi, df = n - 1))
  paste0(
    sprintf("%.2f", mean(z)),
    ifelse(is.finite(mean_p) && mean_p < 0.05, "*", ""),
    " (",
    sprintf("%.2f", sd_z),
    ifelse(is.finite(var_p) && var_p < 0.05, "\u2020", ""),
    ")"
  )
}

format_mean_sd <- function(x, digits = 2) {
  paste0(sprintf(paste0("%.", digits, "f"), mean(x, na.rm = TRUE)), " +/- ", sprintf(paste0("%.", digits, "f"), sd(x, na.rm = TRUE)))
}

squared_correlation_r2 <- function(obs, pred) {
  keep <- is.finite(obs) & is.finite(pred)
  if (sum(keep) < 2) return(NA_real_)
  stats::cor(obs[keep], pred[keep])^2
}

table4_rows <- list()
for (sex_label in c("Female", "Male")) {
  df_sex <- subset(validation2, sex == sex_label)
  for (outcome in c("FEV1", "FVC", "FEV1FVC")) {
    obs <- df_sex[[observed_column(outcome)]]
    for (model_name in names(equations)) {
      spec <- equations[[model_name]][[outcome]]
      pred_vals <- df_sex[[spec$pred]]
      if (outcome == "FEV1FVC") pred_vals <- ratio_pred(pred_vals)
      z_vals <- df_sex[[spec$z]]
      ci <- mse_ci((obs - pred_vals)^2)
      table4_rows[[length(table4_rows) + 1]] <- data.frame(
        Sex = sex_label,
        Outcome = ifelse(outcome == "FEV1FVC", "FEV1/FVC", outcome),
        Model = model_name,
        MSE = ci[1],
        CI_low = ci[2],
        CI_high = ci[3],
        MSE_95CI = sprintf("%.3f [%.3f, %.3f]", ci[1], ci[2], ci[3]),
        R2 = squared_correlation_r2(obs, pred_vals),
        Observed_mean_SD = format_mean_sd(obs),
        Predicted_mean_SD = format_mean_sd(pred_vals),
        Z_score_tests = z_cell(z_vals),
        Below_LLN_percent = 100 * mean(z_vals < q05, na.rm = TRUE),
        stringsAsFactors = FALSE
      )
    }
  }
}
table4 <- do.call(rbind, table4_rows)
write.csv(table4, file.path(script_dir, "Table4_external_validation_development_sigma_lln.csv"), row.names = FALSE)

age_band <- function(age) {
  cut(age, breaks = c(-Inf, 10, 14, Inf), labels = c("<10", "10-<14", ">=14"), right = FALSE)
}
training$age_band <- age_band(training$age_years)
validation2$age_band <- age_band(validation2$age_years)

residual_plot_df <- rbind(
  data.frame(
    Sex = validation2$sex,
    Age_band = validation2$age_band,
    Outcome = "FEV1",
    Residual = validation2$FEV1_L - validation2$pred_FEV1_L
  ),
  data.frame(
    Sex = validation2$sex,
    Age_band = validation2$age_band,
    Outcome = "FVC",
    Residual = validation2$FVC_L - validation2$pred_FVC_L
  ),
  data.frame(
    Sex = validation2$sex,
    Age_band = validation2$age_band,
    Outcome = "FEV1/FVC",
    Residual = validation2$FEV1FVC_ratio_model - validation2$pred_FEV1FVC_ratio
  )
)

make_residual_plot <- function(sex_label, display_label, filename) {
  p <- ggplot(subset(residual_plot_df, Sex == sex_label), aes(x = Age_band, y = Residual)) +
    geom_hline(yintercept = 0, linetype = 2, color = "gray40") +
    geom_boxplot(fill = "white", color = "black", outlier.alpha = 0.4) +
    facet_wrap(~Outcome, scales = "free_y") +
    labs(
      x = "Age band",
      y = "Observed - predicted",
      title = paste("Residuals of final selected models in", display_label)
    ) +
    theme_bw(base_size = 11)
  ggsave(file.path(script_dir, filename), p, width = 7, height = 4.5, dpi = 300)
}

make_residual_plot("Male", "boys", "Figure2_residuals_boys_development_sigma.png")
make_residual_plot("Female", "girls", "Figure3_residuals_girls_development_sigma.png")

age_rows <- list()
for (sex_label in c("Female", "Male")) {
  train_sex <- subset(training, sex == sex_label)
  valid_sex <- subset(validation2, sex == sex_label)
  for (band in levels(training$age_band)) {
    train_n <- sum(train_sex$age_band == band, na.rm = TRUE)
    valid_band <- subset(valid_sex, age_band == band)
    valid_n <- nrow(valid_band)
    for (outcome in c("FEV1", "FVC", "FEV1FVC")) {
      obs <- valid_band[[observed_column(outcome)]]
      pred_col <- if (outcome == "FEV1") "pred_FEV1_L" else if (outcome == "FVC") "pred_FVC_L" else "pred_FEV1FVC_ratio"
      z_col <- if (outcome == "FEV1") "z_FEV1" else if (outcome == "FVC") "z_FVC" else "z_FEV1FVC"
      pred_vals <- valid_band[[pred_col]]
      z_vals <- valid_band[[z_col]]
      age_rows[[length(age_rows) + 1]] <- data.frame(
        Sex = sex_label,
        Age_band = band,
        Training_n_percent = sprintf("%d (%.1f)", train_n, 100 * train_n / nrow(train_sex)),
        Validation_n_percent = sprintf("%d (%.1f)", valid_n, 100 * valid_n / nrow(valid_sex)),
        Outcome = ifelse(outcome == "FEV1FVC", "FEV1/FVC", outcome),
        MSE = mean((obs - pred_vals)^2, na.rm = TRUE),
        MAE = mean(abs(obs - pred_vals), na.rm = TRUE),
        Mean_z_SD = sprintf("%.2f (%.2f)", mean(z_vals, na.rm = TRUE), sd(z_vals, na.rm = TRUE)),
        Below_LLN_percent = 100 * mean(z_vals < q05, na.rm = TRUE),
        stringsAsFactors = FALSE
      )
    }
  }
}
table5 <- do.call(rbind, age_rows)
write.csv(table5, file.path(script_dir, "Table5_age_stratified_validation_development_sigma_lln.csv"), row.names = FALSE)

age_equation_rows <- list()
for (sex_label in c("Female", "Male")) {
  valid_sex <- subset(validation2, sex == sex_label)
  for (band in levels(training$age_band)) {
    valid_band <- subset(valid_sex, age_band == band)
    valid_n <- nrow(valid_band)
    for (outcome in c("FEV1", "FVC", "FEV1FVC")) {
      obs <- valid_band[[observed_column(outcome)]]
      for (model_name in names(equations)) {
        spec <- equations[[model_name]][[outcome]]
        pred_vals <- valid_band[[spec$pred]]
        if (outcome == "FEV1FVC") pred_vals <- ratio_pred(pred_vals)
        z_vals <- valid_band[[spec$z]]
        age_equation_rows[[length(age_equation_rows) + 1]] <- data.frame(
          Sex = sex_label,
          Age_band = band,
          Validation_n = valid_n,
          Outcome = ifelse(outcome == "FEV1FVC", "FEV1/FVC", outcome),
          Model = model_name,
          MSE = mean((obs - pred_vals)^2, na.rm = TRUE),
          MAE = mean(abs(obs - pred_vals), na.rm = TRUE),
          Mean_z = mean(z_vals, na.rm = TRUE),
          SD_z = sd(z_vals, na.rm = TRUE),
          Below_LLN_percent = 100 * mean(z_vals < q05, na.rm = TRUE),
          stringsAsFactors = FALSE
        )
      }
    }
  }
}
age_equation_df <- do.call(rbind, age_equation_rows)
write.csv(age_equation_df, file.path(script_dir, "age_stratified_validation_by_equation_development_sigma_lln.csv"), row.names = FALSE)

age_equation_df$Model <- factor(
  age_equation_df$Model,
  levels = c("Study predictive model", "Al-Qerem", "GLI 2012 Caucasian", "GLI 2022")
)
age_equation_df$Age_band <- factor(age_equation_df$Age_band, levels = c("<10", "10-<14", ">=14"))
equation_dodge <- position_dodge(width = 0.24)

age_plot <- ggplot(
  age_equation_df,
  aes(x = Age_band, y = Below_LLN_percent, group = Model, color = Model, shape = Model, linetype = Model)
) +
  geom_hline(yintercept = 5, linetype = 2, color = "gray35", linewidth = 0.3) +
  geom_line(linewidth = 0.55, position = equation_dodge) +
  geom_point(size = 1.9, position = equation_dodge) +
  facet_grid(Outcome ~ Sex) +
  scale_y_continuous(limits = c(0, NA), expand = expansion(mult = c(0, 0.08))) +
  labs(
    x = "Age band, years",
    y = "Below LLN, %",
    color = "Equation",
    shape = "Equation",
    linetype = "Equation"
  ) +
  theme_bw(base_size = 10) +
  theme(
    legend.position = "bottom",
    legend.box = "vertical",
    panel.grid.minor = element_blank(),
    strip.background = element_rect(fill = "gray92", color = "gray50"),
    axis.text.x = element_text(angle = 0, hjust = 0.5)
  )

ggsave(
  file.path(script_dir, "Figure8_age_stratified_below_LLN_by_equation_development_sigma.png"),
  age_plot,
  width = 7.3,
  height = 5.2,
  dpi = 300
)

below_rows <- list()
for (sex_label in c("Female", "Male")) {
  df_sex <- subset(validation2, sex == sex_label)
  for (model_name in names(equations)) {
    for (outcome in c("FEV1", "FVC", "FEV1FVC")) {
      z_col <- equations[[model_name]][[outcome]]$z
      below_rows[[length(below_rows) + 1]] <- data.frame(
        Sex = sex_label,
        Model = model_name,
        Outcome = ifelse(outcome == "FEV1FVC", "FEV1/FVC", outcome),
        Below_LLN_percent = 100 * mean(df_sex[[z_col]] < q05, na.rm = TRUE),
        stringsAsFactors = FALSE
      )
    }
  }
}
below_df <- do.call(rbind, below_rows)
write.csv(below_df, file.path(script_dir, "below_LLN_by_equation_development_sigma_lln.csv"), row.names = FALSE)

make_below_plot <- function(sex_label, filename) {
  p <- ggplot(subset(below_df, Sex == sex_label), aes(x = Model, y = Below_LLN_percent, fill = Outcome)) +
    geom_col(position = position_dodge(width = 0.8), width = 0.7, color = "black", linewidth = 0.2) +
    coord_flip() +
    labs(x = NULL, y = "Below LLN, %", fill = "Outcome") +
    theme_bw(base_size = 11)
  ggsave(file.path(script_dir, filename), p, width = 7, height = 4.5, dpi = 300)
}

make_below_plot("Male", "Figure4_LLN_boys_development_sigma.png")
make_below_plot("Female", "Figure5_LLN_girls_development_sigma.png")

classify_pattern <- function(z_ratio, z_fvc) {
  ratio_low <- z_ratio < q05
  fvc_low <- z_fvc < q05
  ifelse(
    ratio_low & fvc_low, "Mixed",
    ifelse(ratio_low & !fvc_low, "Obstructive",
           ifelse(!ratio_low & fvc_low, "Restrictive/nonspecific", "Normal"))
  )
}

pattern_rows <- list()
for (sex_label in c("Female", "Male")) {
  df_sex <- subset(validation2, sex == sex_label)
  for (model_name in names(equations)) {
    pattern <- classify_pattern(
      df_sex[[equations[[model_name]]$FEV1FVC$z]],
      df_sex[[equations[[model_name]]$FVC$z]]
    )
    tab <- prop.table(table(factor(pattern, levels = c("Normal", "Obstructive", "Restrictive/nonspecific", "Mixed")))) * 100
    pattern_rows[[length(pattern_rows) + 1]] <- data.frame(
      Sex = sex_label,
      Model = model_name,
      Pattern = names(tab),
      Percent = as.numeric(tab),
      stringsAsFactors = FALSE
    )
  }
}
pattern_df <- do.call(rbind, pattern_rows)
write.csv(pattern_df, file.path(script_dir, "ventilatory_patterns_development_sigma_lln.csv"), row.names = FALSE)

make_pattern_plot <- function(sex_label, filename) {
  p <- ggplot(subset(pattern_df, Sex == sex_label), aes(x = Model, y = Percent, fill = Pattern)) +
    geom_col(color = "black", linewidth = 0.2) +
    coord_flip() +
    labs(x = NULL, y = "Classification, %", fill = "Pattern") +
    theme_bw(base_size = 11)
  ggsave(file.path(script_dir, filename), p, width = 7, height = 4.5, dpi = 300)
}

make_pattern_plot("Male", "Figure6_patterns_boys_development_sigma.png")
make_pattern_plot("Female", "Figure7_patterns_girls_development_sigma.png")

old_below_path <- file.path(script_dir, "below_LLN_by_equation_original_scale_corrected.csv")
if (file.exists(old_below_path)) {
  old_below <- read.csv(old_below_path, stringsAsFactors = FALSE)
  names(old_below)[names(old_below) == "Below_LLN_percent"] <- "Old_Below_LLN_percent"
  names(below_df)[names(below_df) == "Below_LLN_percent"] <- "Development_sigma_Below_LLN_percent"
  comparison <- merge(old_below, below_df, by = c("Sex", "Model", "Outcome"), all = TRUE)
  comparison$Difference_percentage_points <- comparison$Development_sigma_Below_LLN_percent - comparison$Old_Below_LLN_percent
  write.csv(comparison, file.path(script_dir, "below_LLN_comparison_original_vs_development_sigma.csv"), row.names = FALSE)
}

report_lines <- c(
  "Development-data sigma LLN analysis",
  "",
  "Mean models: selected final models from the original-scale candidate-model workflow.",
  "Sigma models: same model class and predictor scale as the selected mean model for each sex-outcome combination.",
  "Sigma training target: log((development out-of-fold residual - residual mean)^2 + eps).",
  "Sigma conversion: sqrt(exp(predicted log variance - E[log(Chi-square_1)])); E[log(Chi-square_1)] = digamma(0.5) + log(2).",
  "Validation data were not used to estimate mean models, sigma models, residual bias, or sigma floors.",
  "GLI 2022 comparator predictions and z-scores were recalculated with rspiro::pred_GLIgl() and rspiro::zscore_GLIgl(); stored GLIgl columns in the input dataset were not used.",
  "External-validation R2 definition: squared Pearson correlation between observed and predicted values on the original clinical measurement scale.",
  "",
  "Study predictive model external-validation summary:",
  paste(capture.output(print(subset(table4, Model == "Study predictive model", select = c("Sex", "Outcome", "MSE_95CI", "R2", "Z_score_tests", "Below_LLN_percent")), row.names = FALSE)), collapse = "\n"),
  "",
  "Files written:",
  "S2_validation_predictions_development_sigma_lln.csv",
  "S2_development_sigma_model_report.csv",
  "S4_fitted_reference_models_development_sigma_lln.rds",
  "Table4_external_validation_development_sigma_lln.csv",
  "Table5_age_stratified_validation_development_sigma_lln.csv",
  "age_stratified_validation_by_equation_development_sigma_lln.csv",
  "Figure1_age_distribution_by_phase_sex.png",
  "Figure2_residuals_boys_development_sigma.png",
  "Figure3_residuals_girls_development_sigma.png",
  "Figure8_age_stratified_below_LLN_by_equation_development_sigma.png",
  "below_LLN_by_equation_development_sigma_lln.csv",
  "below_LLN_comparison_original_vs_development_sigma.csv",
  "Figure4_LLN_boys_development_sigma.png",
  "Figure5_LLN_girls_development_sigma.png",
  "ventilatory_patterns_development_sigma_lln.csv",
  "Figure6_patterns_boys_development_sigma.png",
  "Figure7_patterns_girls_development_sigma.png"
)

writeLines(report_lines, file.path(script_dir, "development_sigma_lln_analysis_report.txt"))

message("Completed development-data sigma LLN workflow. Outputs written to: ", script_dir)
```

## D. Supplementary lower-tail quantile regression for FEV1/FVC z-scores

```
required_packages <- c("quantreg", "rspiro")
missing <- required_packages[!vapply(required_packages, requireNamespace, logical(1), quietly = TRUE)]
if (length(missing) > 0) stop("Missing packages: ", paste(missing, collapse = ", "))

suppressPackageStartupMessages({
  library(quantreg)
  library(rspiro)
})

base_dir <- "C:/Users/LENOVO/OneDrive/Latest stuudies/GLI Global/children/Machine learning-children"
submission_dir <- file.path(base_dir, "submission")
out_dir <- file.path(base_dir, "june 1st")

dat <- read.csv(file.path(submission_dir, "S1_deidentified_analysis_dataset_children.csv"), stringsAsFactors = FALSE)
pred <- read.csv(file.path(submission_dir, "S2_validation_predictions_development_sigma_lln.csv"), stringsAsFactors = FALSE)

dat <- subset(dat, analysis_split == "external_validation")
dat$FEV1FVC_ratio_model <- as.numeric(dat$FEV1FVC_ratio)

gli_global_gender <- ifelse(dat$sex == "Male", 1, ifelse(dat$sex == "Female", 2, NA_real_))
gli_global_z <- zscore_GLIgl(
  age = dat$age_years,
  height = dat$height_cm / 100,
  gender = gli_global_gender,
  FEV1 = dat$FEV1_L,
  FVC = dat$FVC_L,
  FEV1FVC = dat$FEV1FVC_ratio_model
)
dat$GLIgl_FEV1_z <- gli_global_z$z.score.FEV1
dat$GLIgl_FVC_z <- gli_global_z$z.score.FVC
dat$GLIgl_FEV1FVC_z <- gli_global_z$z.score.FEV1FVC

pred_keep <- pred[, c("participant_id", "z_FEV1", "z_FVC", "z_FEV1FVC")]
dat <- merge(dat, pred_keep, by = "participant_id", all.x = TRUE)

z_map <- data.frame(
  equation = c(rep("Study predictive model", 3), rep("Al-Qerem", 3), rep("GLI 2012 Caucasian", 3), rep("GLI 2022", 3)),
  outcome = rep(c("FEV1", "FVC", "FEV1/FVC"), 4),
  z_col = c(
    "z_FEV1", "z_FVC", "z_FEV1FVC",
    "ALQ_FEV1_z", "ALQ_FVC_z", "ALQ_FEV1FVC_z",
    "GLI2012_FEV1_z", "GLI2012_FVC_z", "GLI2012_FEV1FVC_z",
    "GLIgl_FEV1_z", "GLIgl_FVC_z", "GLIgl_FEV1FVC_z"
  ),
  stringsAsFactors = FALSE
)

coef_rows <- list()
model_rows <- list()
get_lm_coef <- function(fit, term) {
  sm <- summary(fit)$coefficients
  c(estimate = sm[term, "Estimate"], se = sm[term, "Std. Error"], p = sm[term, "Pr(>|t|)"])
}
get_rq_coef <- function(fit, term) {
  sm <- tryCatch(summary(fit, se = "nid")$coefficients, error = function(e) NULL)
  if (is.null(sm) || !(term %in% rownames(sm))) return(c(estimate = NA_real_, se = NA_real_, p = NA_real_))
  pcol <- grep("Pr", colnames(sm), value = TRUE)[1]
  c(estimate = sm[term, "Value"], se = sm[term, "Std. Error"], p = sm[term, pcol])
}

for (sex_i in c("Female", "Male")) {
  for (i in seq_len(nrow(z_map))) {
    z_col <- z_map$z_col[i]
    d <- dat[dat$sex == sex_i & is.finite(dat[[z_col]]) & is.finite(dat$age_years) & is.finite(dat$height_cm), ]
    d$z <- d[[z_col]]
    d$height_10cm <- d$height_cm / 10
    lm_fit <- lm(z ~ age_years + height_10cm, data = d)
    rq50 <- rq(z ~ age_years + height_10cm, tau = 0.50, data = d)
    rq05 <- rq(z ~ age_years + height_10cm, tau = 0.05, data = d)
    sm_lm <- summary(lm_fit)
    model_rows[[length(model_rows) + 1]] <- data.frame(
      sex = sex_i, equation = z_map$equation[i], outcome = z_map$outcome[i],
      n = nrow(d), mean_z = mean(d$z), sd_z = sd(d$z), lm_r2 = sm_lm$r.squared,
      q05_observed = as.numeric(quantile(d$z, 0.05, na.rm = TRUE)),
      q50_observed = as.numeric(quantile(d$z, 0.50, na.rm = TRUE)),
      stringsAsFactors = FALSE
    )
    for (term in c("age_years", "height_10cm")) {
      lc <- get_lm_coef(lm_fit, term)
      q50c <- get_rq_coef(rq50, term)
      q05c <- get_rq_coef(rq05, term)
      coef_rows[[length(coef_rows) + 1]] <- data.frame(
        sex = sex_i, equation = z_map$equation[i], outcome = z_map$outcome[i],
        method = "Linear mean", tau = NA_real_, term = term,
        estimate = lc["estimate"], se = lc["se"], p = lc["p"], stringsAsFactors = FALSE
      )
      coef_rows[[length(coef_rows) + 1]] <- data.frame(
        sex = sex_i, equation = z_map$equation[i], outcome = z_map$outcome[i],
        method = "Quantile median", tau = 0.50, term = term,
        estimate = q50c["estimate"], se = q50c["se"], p = q50c["p"], stringsAsFactors = FALSE
      )
      coef_rows[[length(coef_rows) + 1]] <- data.frame(
        sex = sex_i, equation = z_map$equation[i], outcome = z_map$outcome[i],
        method = "Quantile lower tail", tau = 0.05, term = term,
        estimate = q05c["estimate"], se = q05c["se"], p = q05c["p"], stringsAsFactors = FALSE
      )
    }
  }
}

model_summary <- do.call(rbind, model_rows)
coef_summary <- do.call(rbind, coef_rows)
coef_summary$term_label <- ifelse(coef_summary$term == "age_years", "Age, per year", "Height, per 10 cm")
coef_summary$significant <- is.finite(coef_summary$p) & coef_summary$p < 0.05

write.csv(model_summary, file.path(out_dir, "zscore_age_height_model_summary.csv"), row.names = FALSE)
write.csv(coef_summary, file.path(out_dir, "zscore_age_height_regression_coefficients.csv"), row.names = FALSE)

sig_counts <- aggregate(significant ~ equation + outcome + method, data = coef_summary, FUN = sum)
names(sig_counts)[names(sig_counts) == "significant"] <- "n_significant_predictors"
write.csv(sig_counts, file.path(out_dir, "zscore_age_height_significance_counts.csv"), row.names = FALSE)

cat("MODEL_SUMMARY\n")
print(model_summary)
cat("\nSIGNIFICANCE_COUNTS\n")
print(sig_counts)
cat("\nCOEFFICIENT_FILE\n", file.path(out_dir, "zscore_age_height_regression_coefficients.csv"), "\n", sep = "")
```

## E. R session information

```
R version 4.5.3 (2026-03-11 ucrt)
Platform: x86_64-w64-mingw32/x64
Running under: Windows 11 x64 (build 26200)

Matrix products: default
  LAPACK version 3.12.1

locale:
[1] LC_COLLATE=English_United States.utf8
[2] LC_CTYPE=English_United States.utf8
[3] LC_MONETARY=English_United States.utf8
[4] LC_NUMERIC=C
[5] LC_TIME=English_United States.utf8

time zone: Asia/Amman
tzcode source: internal

attached base packages:
[1] stats     graphics  grDevices utils     datasets  methods   base

other attached packages:
[1] rspiro_0.5           kernlab_0.9-33       randomForest_4.7-1.2
[4] gbm_2.2.3            caret_7.0-1          lattice_0.22-9
[7] ggplot2_4.0.1

loaded via a namespace (and not attached):
 [1] future_1.69.0        generics_0.1.4       class_7.3-23
 [4] stringi_1.8.7        pROC_1.19.0.1        listenv_0.10.0
 [7] digest_0.6.39        magrittr_2.0.4       grid_4.5.3
[10] timechange_0.3.0     RColorBrewer_1.1-3   iterators_1.0.14
[13] foreach_1.5.2        plyr_1.8.9           Matrix_1.7-4
[16] ModelMetrics_1.2.2.2 nnet_7.3-20          survival_3.8-6
[19] purrr_1.2.0          scales_1.4.0         textshaping_1.0.4
[22] codetools_0.2-20     lava_1.8.2           cli_3.6.5
[25] rlang_1.1.6          hardhat_1.4.2        parallelly_1.46.1
[28] future.apply_1.20.2  splines_4.5.3        withr_3.0.2
[31] prodlim_2025.04.28   tools_4.5.3          parallel_4.5.3
[34] reshape2_1.4.5       dplyr_1.1.4          recipes_1.3.1
[37] globals_0.19.0       vctrs_0.6.5          R6_2.6.1
[40] rpart_4.1.24         stats4_4.5.3         lifecycle_1.0.4
[43] lubridate_1.9.4      stringr_1.6.0        MASS_7.3-65
[46] ragg_1.5.0           pkgconfig_2.0.3      pillar_1.11.1
[49] gtable_0.3.6         glue_1.8.0           data.table_1.18.0
[52] Rcpp_1.1.0           systemfonts_1.3.1    tibble_3.3.0
[55] tidyselect_1.2.1     farver_2.1.2         nlme_3.1-168
[58] labeling_0.4.3       ipred_0.9-15         timeDate_4051.111
[61] gower_1.0.2          compiler_4.5.3       S7_0.2.1
```
